# Supplementary figures and images for: Prion diseases disrupt glutamate/glutamine metabolism in skeletal muscle
Source: PLoS Pathog. 2024 Sep 11;20(9):e1012552. doi: 10.1371/journal.ppat.1012552 (PMC11419395; doi:10.1371/journal.ppat.1012552)

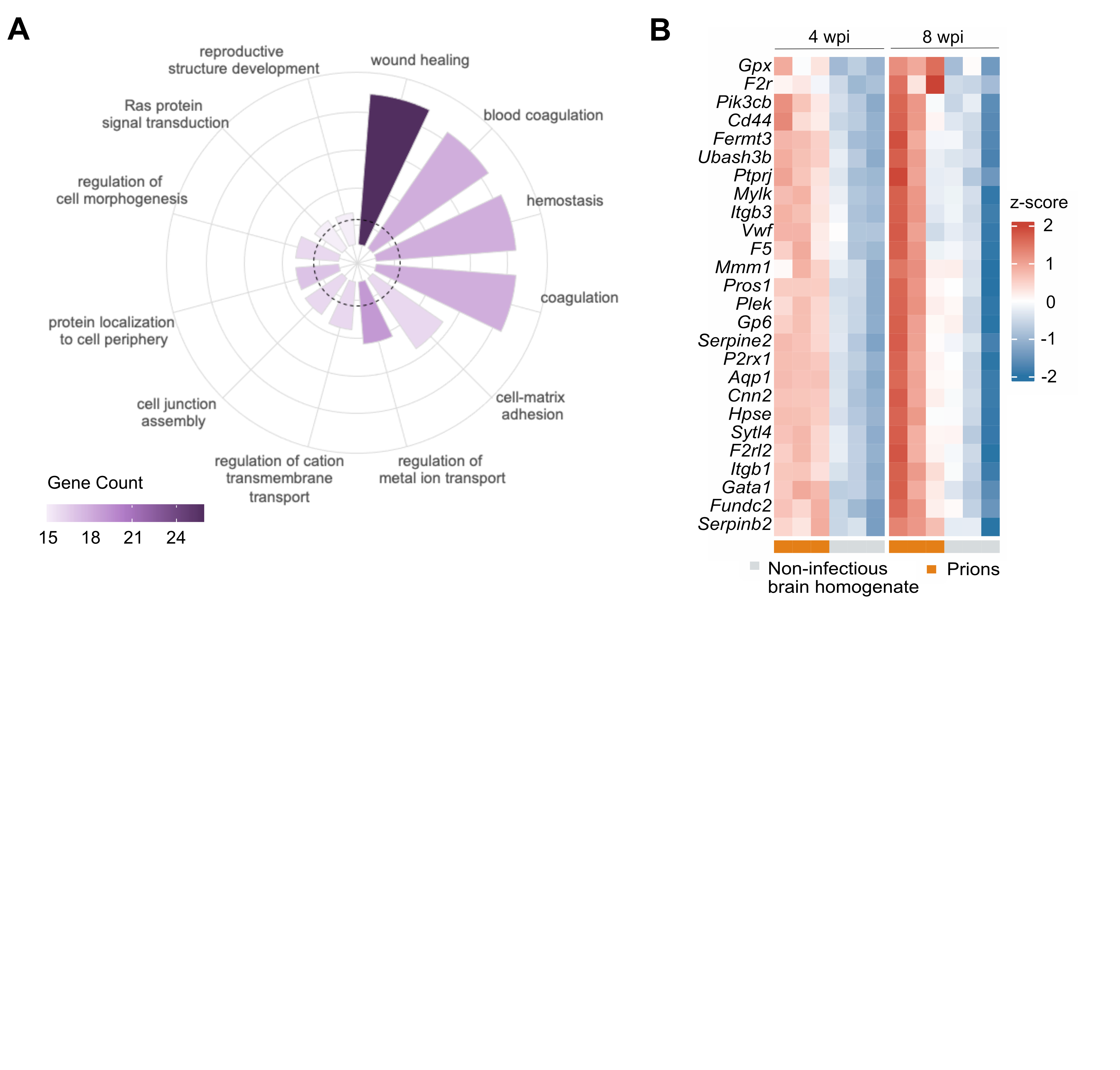

Supplement: S1 Fig — (A) The 270 overlapped, upregulated, blood-derived DEGs at 4wpi and 8 wpi are associated with specific Gene Ontology (GO) terms. The Randarplot displays the results of the GO over-representation analysis by Biological Process (BP) ontology class. (B) Expression patterns (z-score based) of genes related to hemostasis process in blood at 4 and 8 wpi. (TIF) [file ppat.1012552.s001.tif]

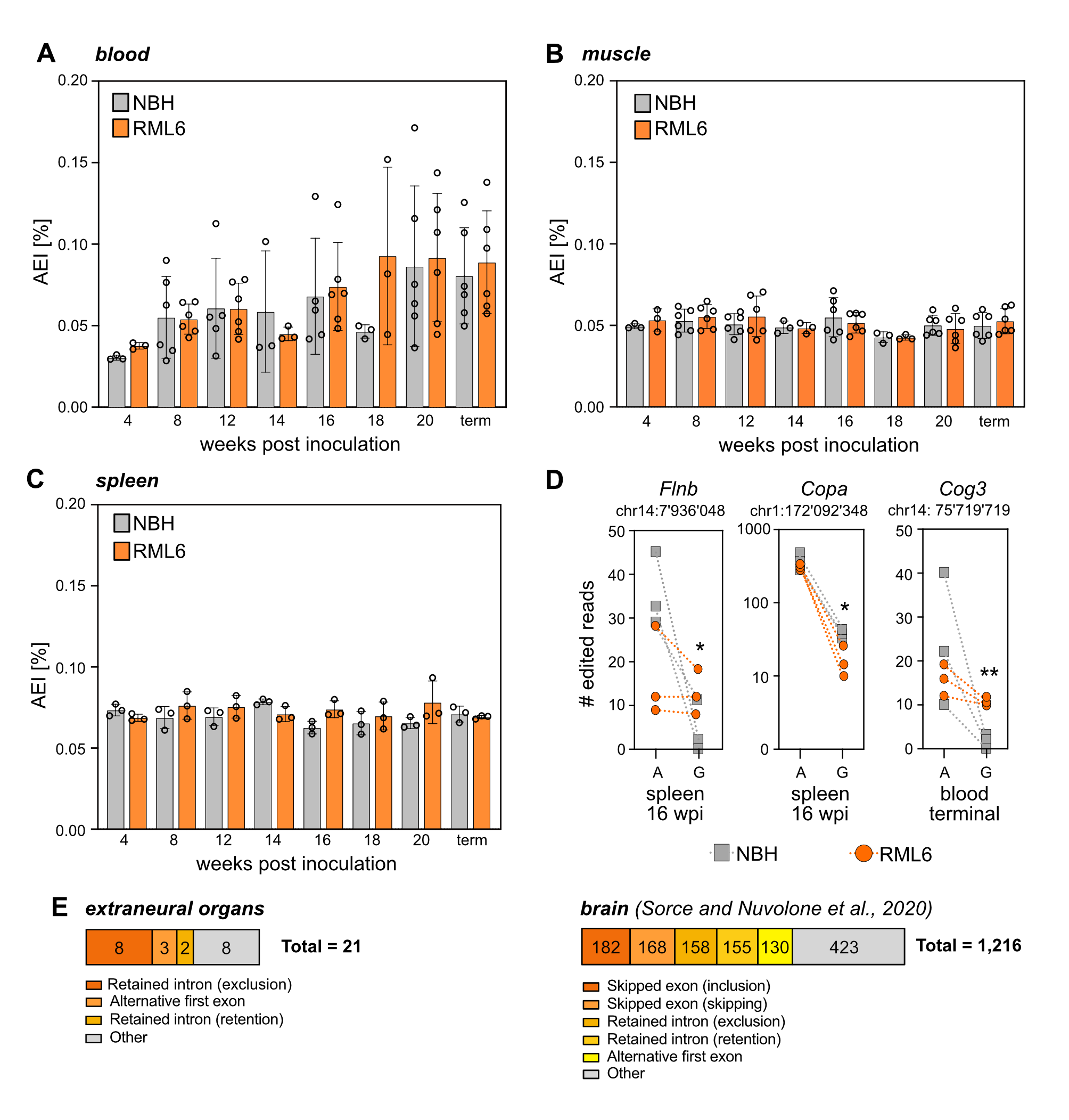

Supplement: S2 Fig — Percentage of alternative splicing events (AEI) in blood (A), muscle (B), and spleen (C) samples of prion-infected (RML6) and control (NBH) mice at various time points post-inoculation. Ordinate: AEI percentage; abscissa: weeks post-inoculation. (D) Significantly recoded gene transcripts Flnb and Copa genes in spleen at 16 wpi. Reduced A-to-I editing of Cog3 transcripts in blood at terminal stage. (E) Bars show annotation and related number of splice variants in both extraneural organs and brains computed from previously reported data [31]. (TIF) [file ppat.1012552.s002.tif]

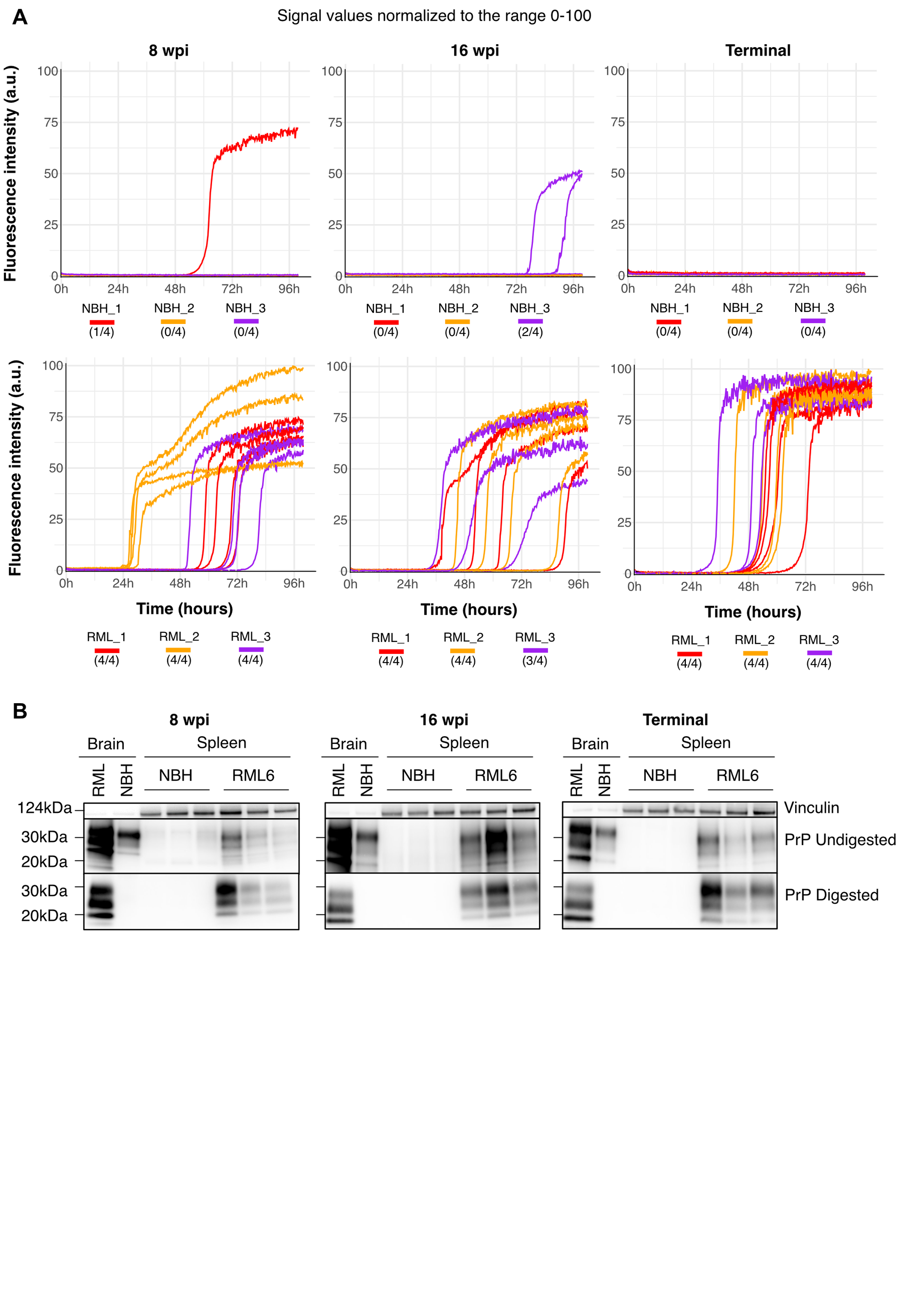

Supplement: S3 Fig — (A) RT-QuIC reactions on spleen homogenates from prion-inoculated mice (RML labeled) and related control (NBH labeled) sacrificed at specific time points. Each sample was tested in quadruplicate, and each plot represents data from biological replicates (n = 3, red, yellow and purple). Ordinate: fluorescent intensity (relative fluorescence units normalized by minimum and maximum value from NBH and RML from the same timepoint). Biological replicates were considered prion positive if 3 out of 4 technical replicates tested positive. (B) Western Blot analysis of spleen before (lane above: PrP Undigested) and after (lane below: PrP Digested) PK treatment. Undigested blot was probed with anti-PrP antibody (POM2). Digested blot was probed with a different anti-PrP antibody (POM1). Brain homogenates were used as controls. (TIF) [file ppat.1012552.s003.tif]

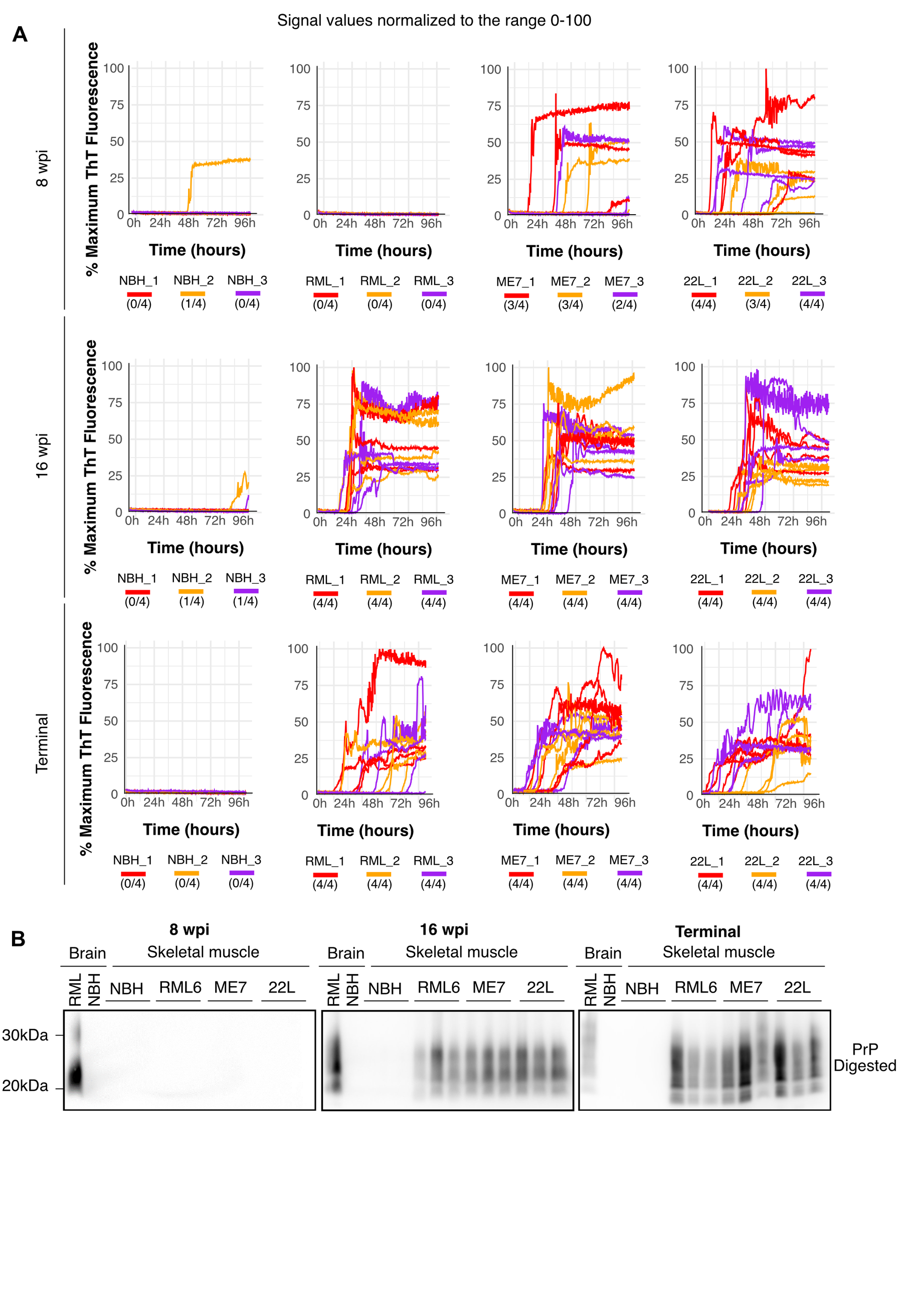

Supplement: S4 Fig — (A) RT-QuIC reactions performed on skeletal muscle derived, NaPTA enriched homogenates from prion-inoculated mice (RML6, ME7 and 22L labeled) and related control (NBH labeled) sacrificed at specific timepoints. Each sample was tested in quadruplicate, and each plot represents data from individual biological replicates (n = 3; red, yellow and purple). Fluorescent intensity on the y axis stands for relative fluorescence units normalized by minimum and maximum value (from control and condition) to obtain percentage. Biological replicates are considered prion positive if 3 out of 4 technical replicates test positive. (B) Western Blot analysis of skeletal muscle after PK treatment. Digested blot is probed with anti-PrP antibody (POM1). Mice infected with different prion strain (RML6, ME7 and 22L) are shown with appropriate segment above the blot. Brain homogenates were used as controls. (TIF) [file ppat.1012552.s004.tif]

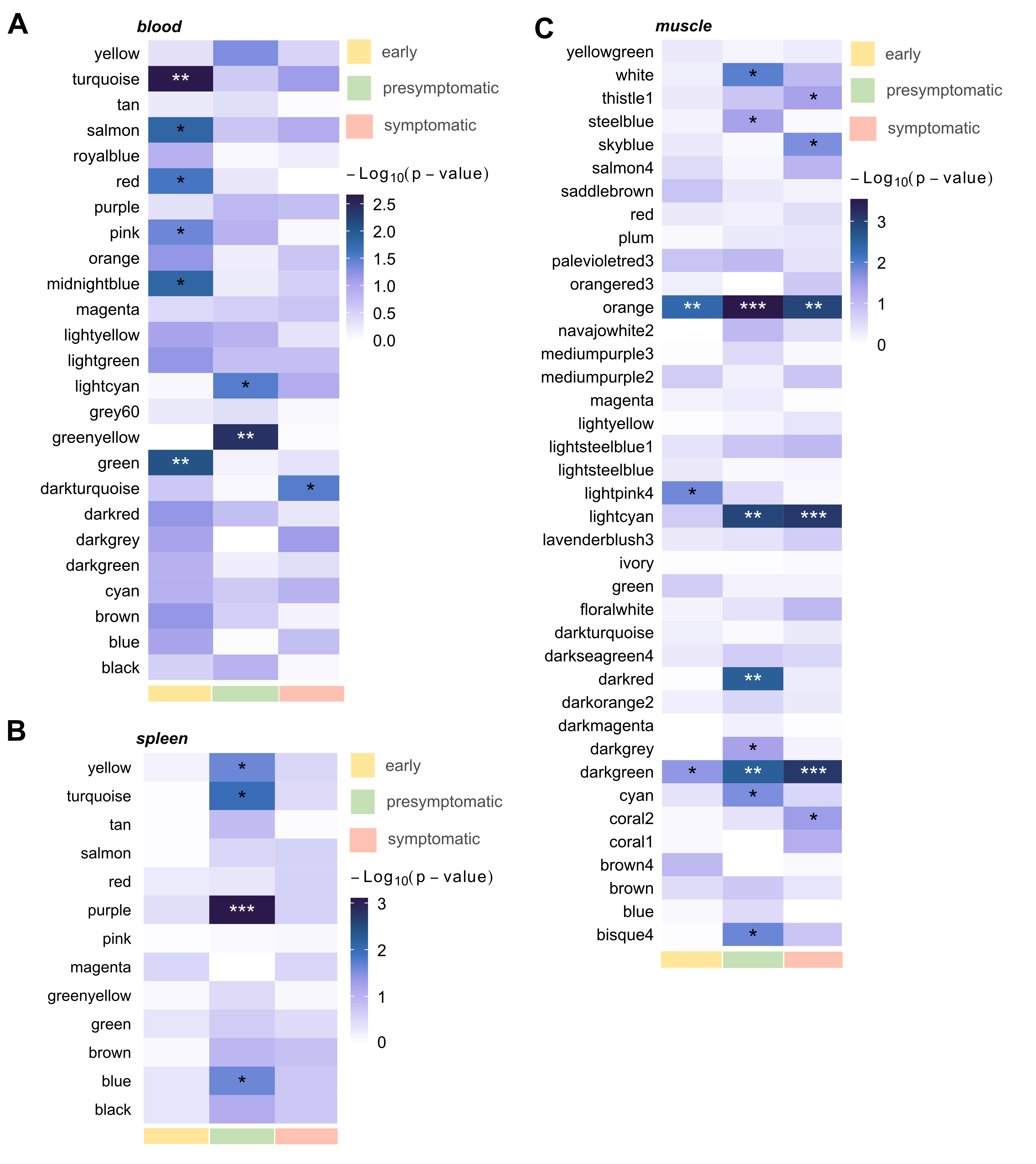

Supplement: S5 Fig — Each heatmap illustrates module eigengene significance at three timestages (early, pre-symptomatic, and symptomatic) derived from the comparison between NBH and RML6 inoculated mice from (A) whole blood, (B) spleen, and (C) skeletal muscles necropsies. Each row represents a specific module, while columns correspond to individual timestages. Statistical significance (*p < 0.05, **p < 0.01, ***p < 0.005, ****p < 0.001) is indicated by asterisks. (TIF) [file ppat.1012552.s005.tif]

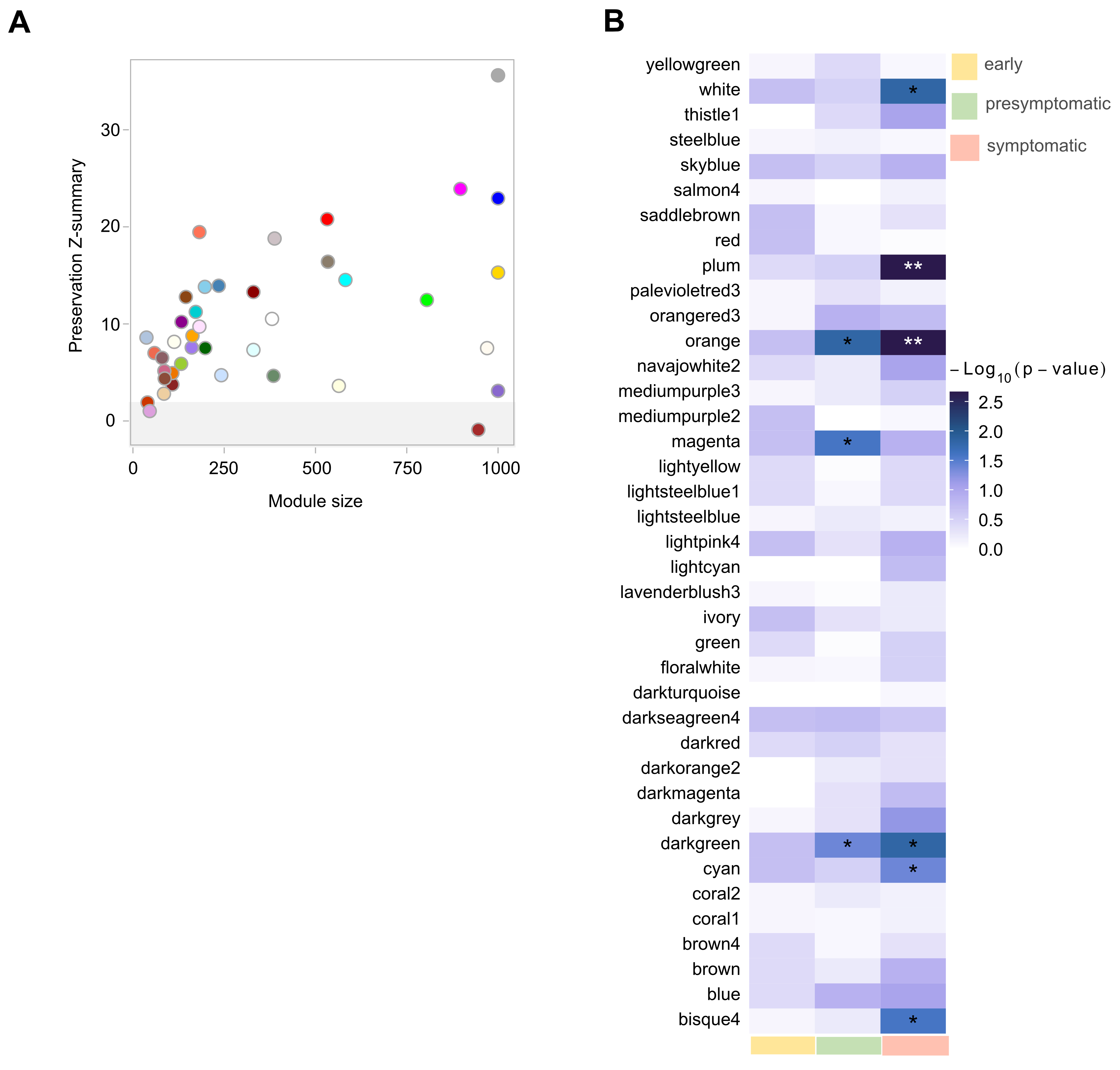

Supplement: S6 Fig — (A) Scatter plot of the Z-summary module preservation statistic and the sizes of modules in muscle co-expression network. The modules with Z-summary > 1.96 were interpreted as preserved. (B) The heatmap illustrates module eigengene significance at three timestages (early, pre-symptomatic and symptomatic) derived from the comparison between NBH and RML6 inoculated mice. Each row represents a specific module, while columns correspond to individual timestages. Statistical significance (*p < 0.05, **p < 0.01, ***p < 0.005, ****p < 0.001) is indicated by asterisks. (TIF) [file ppat.1012552.s006.tif]

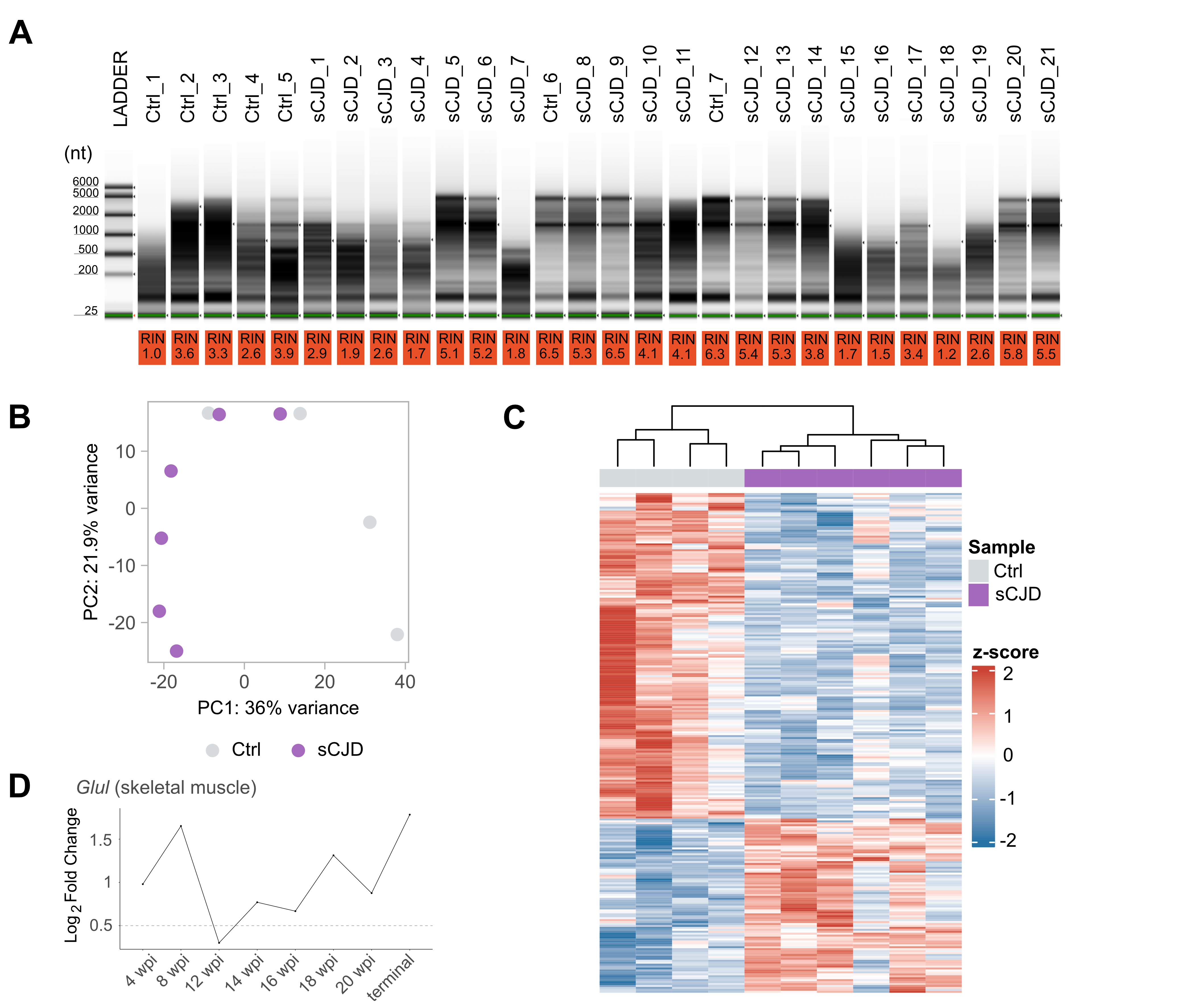

Supplement: S7 Fig — (A) Agilent Bioanalyzer gel image depicting total RNA samples extracted from skeletal muscle tissues of both sCJD patients and control subjects. The image showcases the RNA quality assessment using the Ribosomal Integrity Number (RIN) scores, displayed alongside the respective samples. (B) Principal Component Analysis (PCA) plot illustrating the segregation of gene expression profiles in skeletal muscle samples between individuals with sCJD and non-sCJD controls. Each data point represents a distinct sample, with colors corresponding to two sample conditions. (C) Heatmap illustrating the variation in gene expression between individuals with sCJD and non-sCJD controls. Each row corresponds to a differentially expressed gene, while each column represents an individual subject from either the sCJD or control group. (D) Log2 fold change of Glul transcript derived from the comparison between RML6-infected and NBH treated animals at different analyzed timepoints in skeletal muscle. (TIF) [file ppat.1012552.s007.tif]

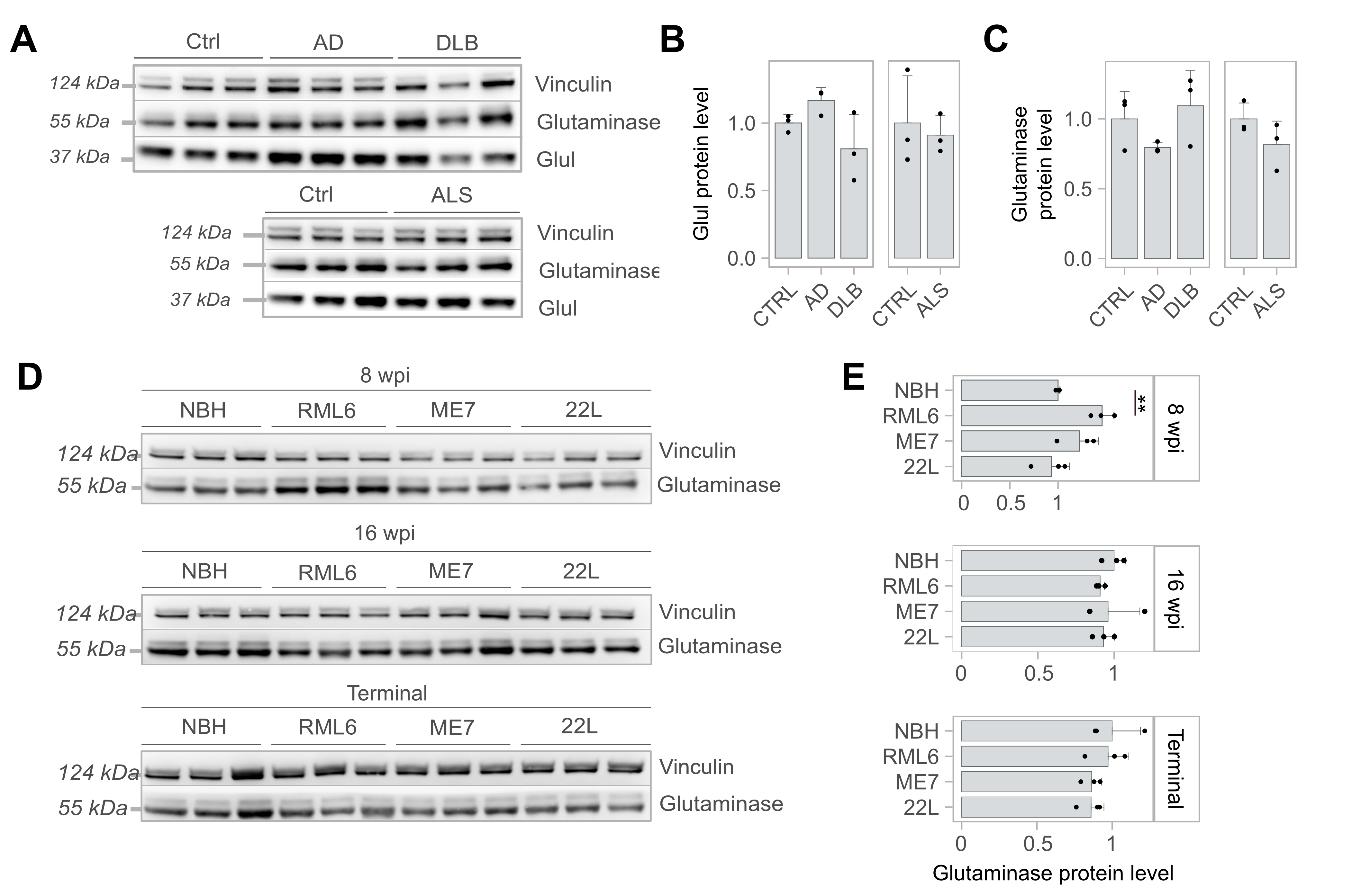

Supplement: S8 Fig — In panel (A) Western blots of Glul, Glutaminase and Vinculin protein levels, of mouse models for AD, DLB and ALS, as well as related control (C57BL/6J). This Western blot presents analysis of two distinct control groups as obtained directly from different collaborators. (B) Densitometry data (ADU) quantification of Glul (normalized with related Vinculin) from the Western blot in S8A Fig. (C) Densitometry data (ADU) quantification of Glutaminase (normalized with related Vinculin) from the Western blot in S8A. Each lane in the Western Blots represents a biological replicate. Statistical significance (*p < 0.05, **p < 0.01, ***p < 0.005, ****p < 0.001) is indicated by asterisks. (D) Western blot of Glutaminase and Vinculin protein of mice inoculated with prion strains RML6, ME7, and 22L, as well as related control (NBH). (E) Densitometry (arbitrary densitometry unit, ADU) quantification of the Western blot in S8D Fig. (TIF) [file ppat.1012552.s008.tif]

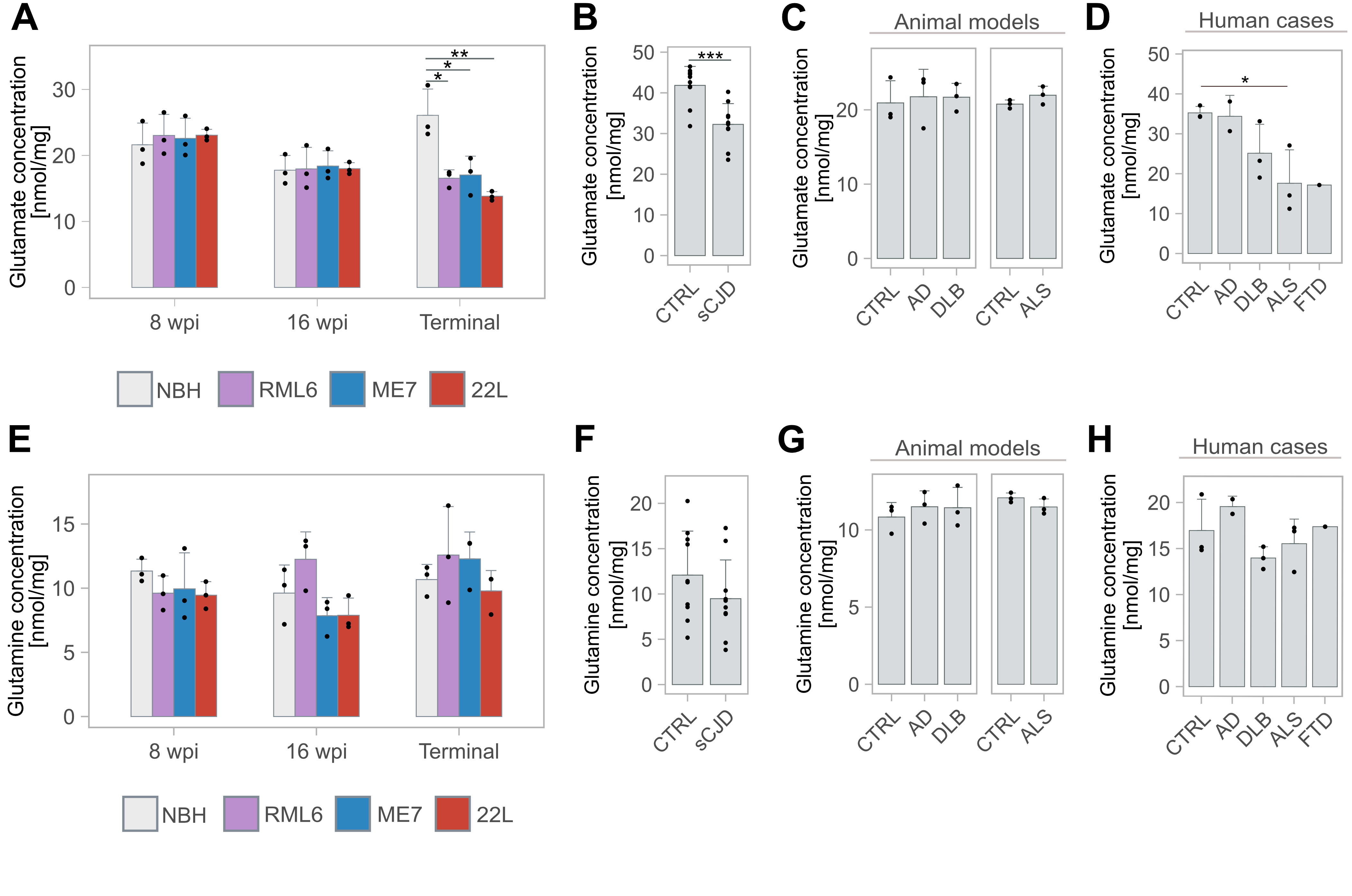

Supplement: S9 Fig — (A) Glutamate concentrations (nmol/mg) in skeletal muscle lysates of (A) mice inoculated with prion strains RML6, ME7, and 22L, as well as related control (NBH) at different timepoints, (B) of sCJD and non-sCJD control patients (C) mouse models of AD, DLB and ALS, as well as related control (C57BL/6J) and (D) human cases of AD, DLB, ALS and FTD. Glutamine concentrations (nmol/mg) in skeletal muscle lysates of (E) mice inoculated with prion strains RML6, ME7, and 22L, as well as related control (NBH) at different timepoints, (F) of sCJD and non-sCJD control patients (G) mouse models of AD, DLB and ALS, as well as related control (C57BL/6J) and (H) human cases of AD, DLB, ALS and FTD. Each dot in the graphs represents a biological replicate. Statistical significance (*p < 0.05, **p < 0.01, ***p < 0.005, ****p < 0.001) is indicated by asterisks. (TIF) [file ppat.1012552.s009.tif]

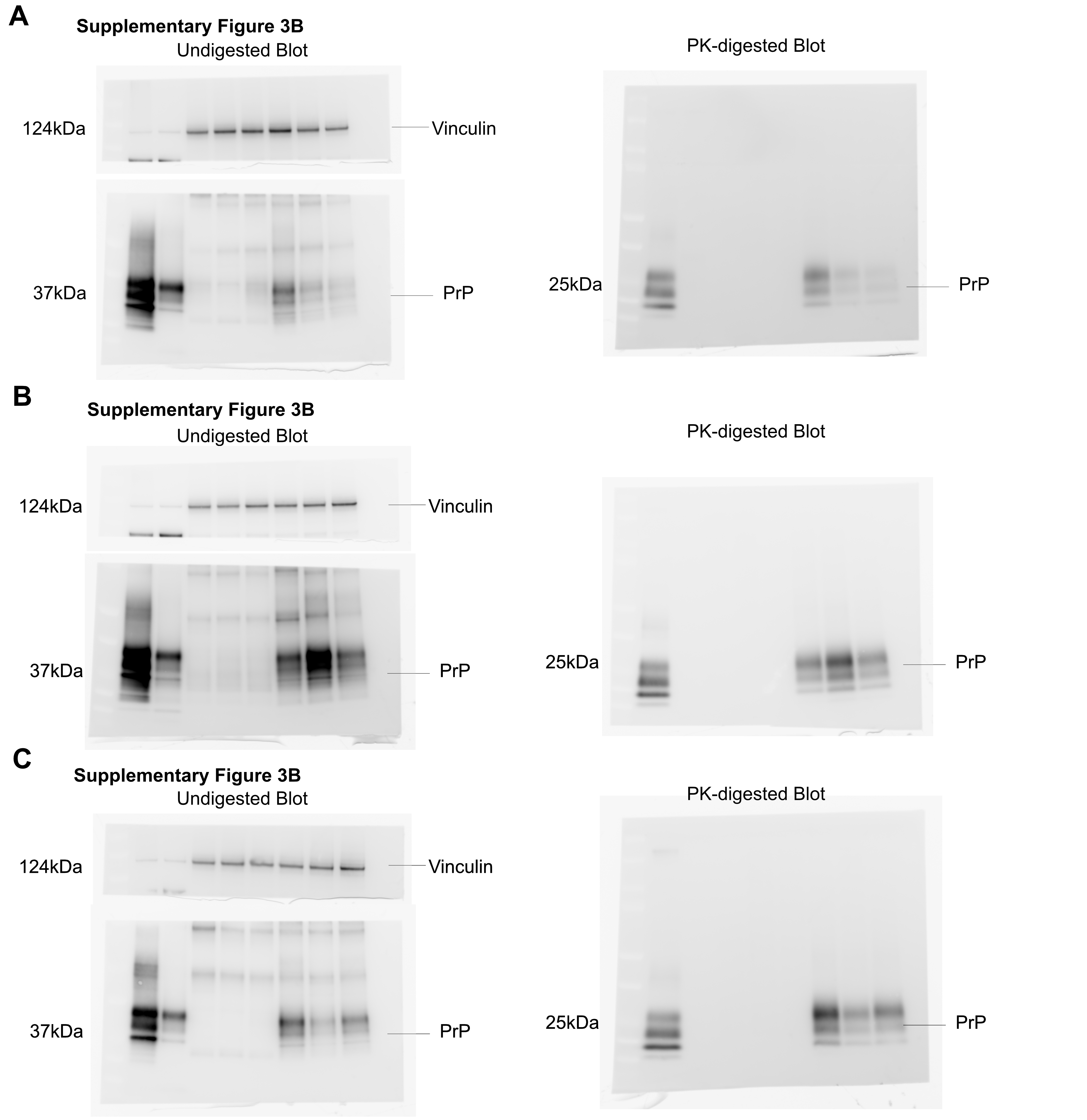

Supplement: S10 Fig — The undigested Western blots were sectioned into two parts, each stained with a different antibody: anti-Vinculin and anti-PrP. Samples represent spleen of infected mice with RML6 prion strain and related NBH control sacrificed at (A) 8 wpi, (B) 16 wpi and (C) terminal stage. (TIF) [file ppat.1012552.s010.tif]

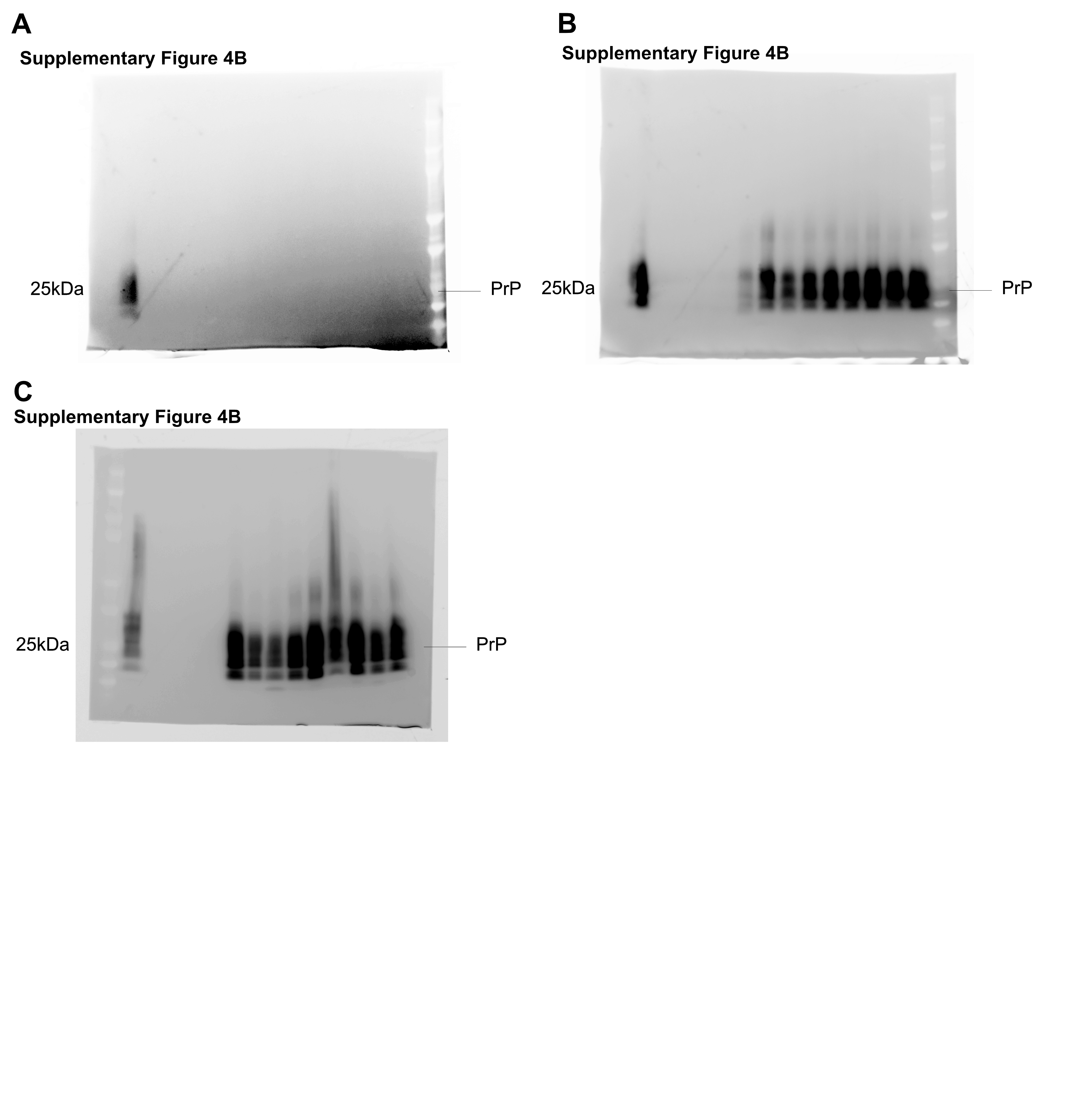

Supplement: S11 Fig — The Western blots were stained with anti-PrP antibody. Samples represent skeletal muscle of infected mice with different prion strains (RML6, ME7 and 22L) and related NBH control sacrificed at (A) 8 wpi, (B) 16 wpi and (C) terminal stage. (TIF) [file ppat.1012552.s011.tif]

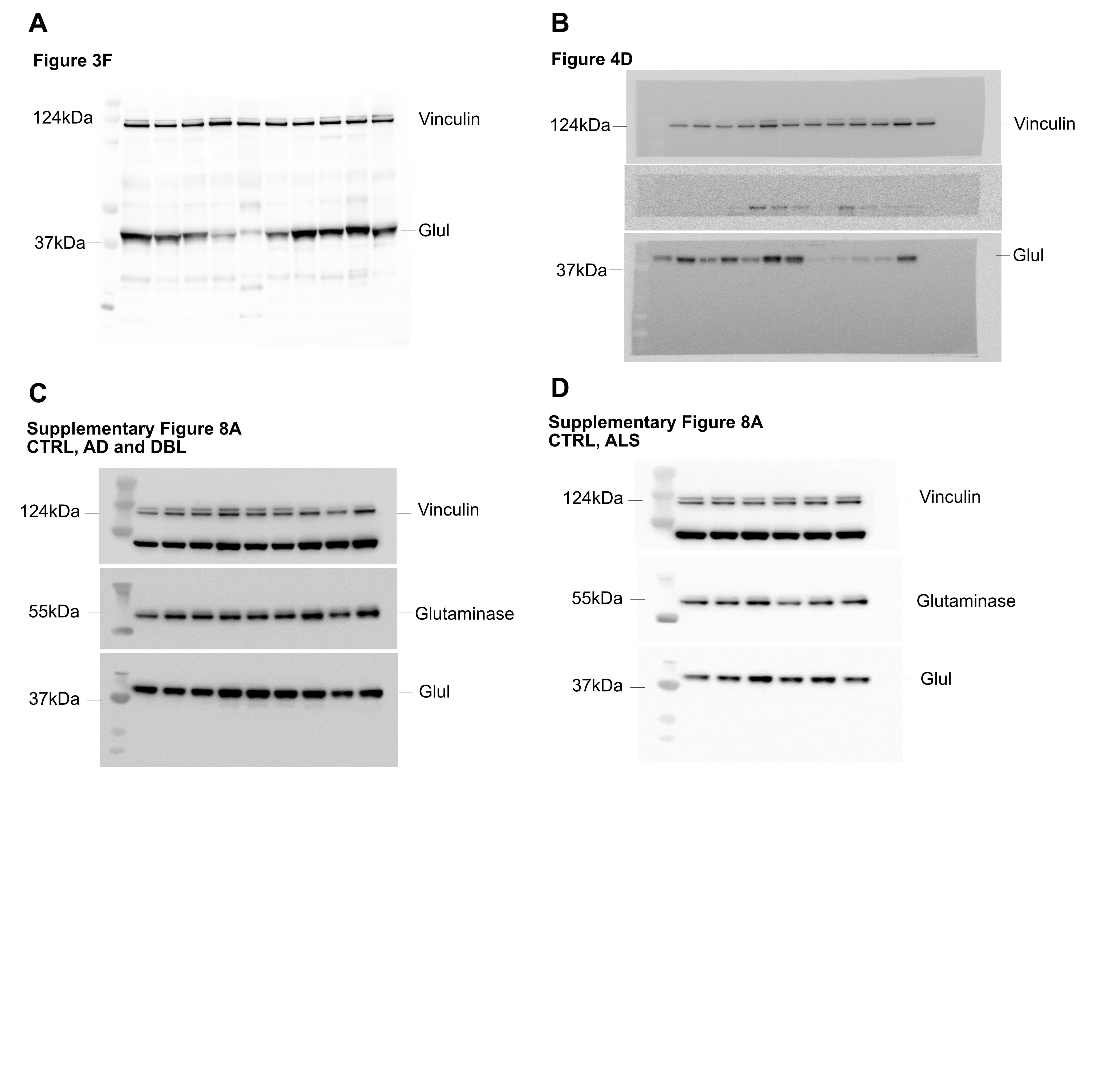

Supplement: S12 Fig — (A) Co-staining of anti-Vinculin and anti-GLUL antibodies on a single blot. (B) Western blot strips were cut from the same gel, divided into three parts for staining with different antibodies (anti-Vinculin and anti-GLUL). The middle section was not used in this paper. (C-D) Western blot strips were cut from the same gel, divided into three parts for staining with different antibodies: anti-Vinculin, anti-Glutaminase and anti-Glul. (TIF) [file ppat.1012552.s012.tif]

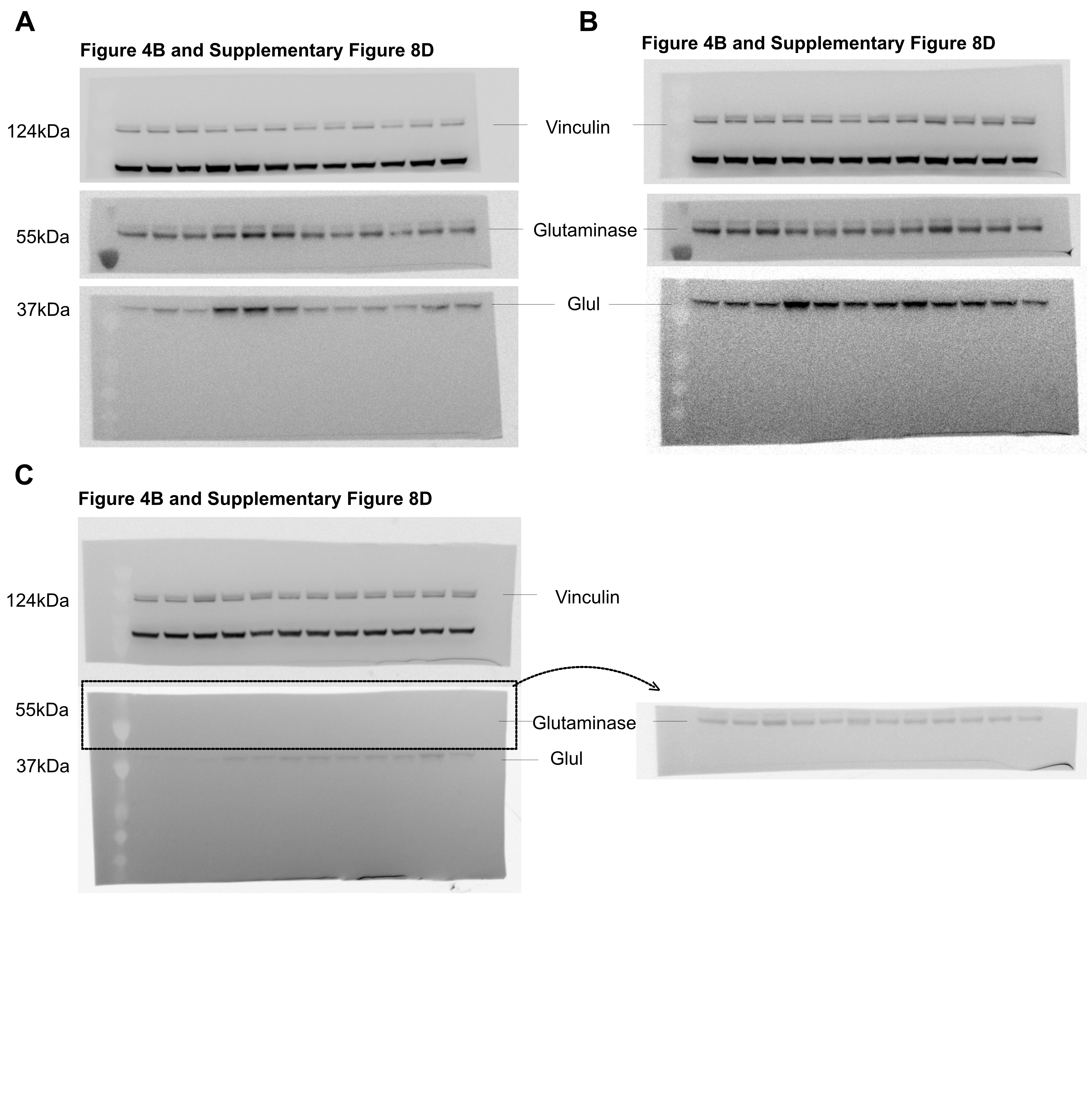

Supplement: S13 Fig — Samples represent skeletal muscle of infected mice with different prion strains (RML6, ME7 and 22L) and related NBH control sacrificed at (A) 8 wpi, (B) 16 wpi and (C) terminal stage. In the (C) panel, the strip intended for Glutaminase protein staining was cut, stained, and acquired after the Glul staining was completed. (TIF) [file ppat.1012552.s013.tif]
