# Supplementary material for: Prion diseases disrupt glutamate/glutamine metabolism in skeletal muscle
Source: PLoS Pathog. 2024 Sep 11;20(9):e1012552. doi: 10.1371/journal.ppat.1012552 (PMC11419395; doi:10.1371/journal.ppat.1012552)
Supplement: Attachment — Submitted filename: Caredio2024_Rebuttal_PLoSPathogens.pdf [file ppat.1012552.s024.pdf]

Full Revision

Review COMMONS PLOS PATHOGENS

Manuscript number: PPATHOGENS-D-24-01272
Corresponding author(s): Adriano, Aguzzi

General Statements

In this study, our primary objective was to investigate the pathophysiological changes occurring in various extraneural organs during prion disease progression. Our focus was on understanding the broader biological impacts of prion infections, particularly alterations in molecular pathways such as the observed glutamine-glutamate cycle. We expected this approach to provide a comprehensive understanding of the disease's systemic effects and identifies potential biomarkers and therapeutic targets relevant to prion diseases.

Our study has generated a FAIR-compliant (Findable, Accessible, Interoperable, Reusable) longitudinal atlas of transcriptional alterations in the CNS level and in an extensive ensemble of extraneural organs. This broader perspective is crucial for developing a more holistic understanding of prion diseases. To facilitate interactive exploration of the results presented in this study and to integrate them with our previously reported findings (1), we have created a searchable database of gene expression profiles from the brain and extraneural organs. This database is available for visualization and download at https://fgcz-shiny.uzh.ch/priontranscriptomics/.

Point-by-point description of the revisions

All the revisions and changes applied to the original manuscript are highlighted in green in the Revised manuscript marked-up copy.

Reviewer #1 Comment:

Absence of evidence demonstrating the presence of prions or prion-seeding activity in extraneural tissues.

Response: Our study focuses on *all* pathophysiological alterations brought about by prion infections in extraneural organs, be they caused directly by prion infections of said organs or through indirect mechanisms. We feel that this agnostic approach is justified, as prion-caused damage is not necessarily/always directly due to prion deposition. Nevertheless, we share the

reviewer's curiosity about a possible correlation between these changes and the presence of prion-seeding activity. We included additional data demonstrating prion presence and seeding activity in spleen and skeletal muscle at various timepoints. This was achieved by employing proteinase K digestion followed by Western blot (PK-WB) and real-time quaking-induced conversion (RT-QuIC) assays to provide a robust correlation with our transcriptomic analyses and Glul upregulation.

We described this in a new Result paragraph of the revised article named *'Prion Deposits Are Found in Spleen and Skeletal Muscles at All Time Points, with Early Absence in Skeletal Muscles of Mice Infected with RML and Presence in ME7 and 22L Strains'*. Furthermore, we have included three related protocols for prion detection assays in Material and Methods section of the revised manuscript: *'Prions detection in the spleen'*, *'Prions detection with NaPTA protocol in the skeletal muscle'*, and *'RT-QuiC protocol'*.

Revision:

Result 'New section'- *'Prion Deposits Are Found in Spleen and Skeletal Muscles at All Time Points, with Early Absence in Skeletal Muscles of Mice Infected with RML and Presence in ME7 and 22L Strains'*.

To determine whether these molecular changes are directly caused by on-site prion deposits, we conducted detection assays at various time points using PK-WB and RT-QuIC assays. We used organs from the same RML prion-infected mice and corresponding NBH control mice previously used for bulk RNA sequencing, selecting a single time point from each stage: 8 wpi for the early stage, 16 wpi for the presymptomatic stage, and the terminal stage for the symptomatic stage. Blood was excluded from this analysis due to hemolysis during RNA sequencing, which made it impossible to separate and collect plasma for RT-QuIC. Whole blood was unsuitable for analysis because blood cells and their products inhibit the RT-QuIC seeding response (2, 3), increasing the likelihood of false negatives. Additionally, blood-derived prions cannot be detected via PK-WB due to low titers.

As shown by other groups (4-6), the spleen accumulated prions early in intracerebrally inoculated mice, despite low PrP^C expression, and the infectious agent load remained constant throughout disease progression (Supplementary Fig. 3, A and B).

The concentration of prions in skeletal muscles is reported to be 10,000 times lower than in the brain, making detection challenging (7, 8). To address this limitation, we used the sodium phosphotungstate anion (NaPTA) reagent with magnesium chloride ($MgCl_2$) to precipitate prions (9). Consistent with other studies (4, 10), we detected prions at 16 wpi and at the terminal stage.

However, prion deposits were not observed at the early time point (8 wpi) in mice infected with the RML prion strain. In contrast, prions were detected in the skeletal muscles of mice infected with other prion strains (ME7 and 22L) at all analyzed time points (Supplementary Fig. 4, A and B). At 8 wpi, prions were detected in mice infected with the ME7 strain in two out of three biological replicates, whereas all replicates showed prion presence in mice infected with the 22L strain. Notably, the PK-WB assay lacked the sensitivity to detect prions at 8 wpi (Supplementary Fig. 4B), whereas RT-QuIC demonstrated significantly higher sensitivity (Supplementary Fig. 4A).

Material and Methods 'New section' - *Prions detection in the spleen.*

50 mg of spleen tissue was homogenized in 500 mL lysis buffer (0.5% wt/vol sodium deoxycholate and 0.5% vol/vol Nonidet P-40 in PBS) at 5'000 rpm for 5 minutes by using a Precellys24 Sample Homogenizer (LABGENE Scientific SA, BER300P24) and incubated on ice for 20 minutes. The cleared lysates were obtained by centrifugation at 1'000 rcf for 6 minutes in an Eppendorf 5417 R tabletop centrifuge. For PK-WB, the concentration of whole protein was determined using a BCA assay (Thermo Scientific). 100 ug of total protein was digested with 20 μg ml−1 proteinase K for 30 min at 37 °C, then mixed with western blotting loading buffer and boiled for 10 min at 95 °C. The following primary antibodies were used: mouse monoclonal antibody against vinculin (1:5000, Abcam, ab129002); mouse monoclonal antibody against PrP (POM1, 1:5,000, homemade) for prions detection; mouse monoclonal antibody against PrP (POM2, 1:5,000, homemade) for PrP^C detection.

Material and Methods 'New section' - *Prions detection with NaPTA protocol in the skeletal muscle.*

To detect prions in skeletal muscles, we performed sodium phosphotungstic acid (NaPTA) enrichment (9). NaPTA binds and precipitates PrP^{Sc} in the presence of $MgCl_2$, removing contaminants and concentrating PrP^{Sc}. For this protocol, 40 mg of tissue were lysed in 400 μl of 2% sarcosyl-PBS by using Precellys24 Sample Homogenizer (LABGENE Scientific SA, BER300P24) twice, at 5'000 rpm for 5 minutes. To remove gross debris, samples were centrifuged at 80g for 1 minute. Samples were next incubated at 37°C while shaking at 1500 rpm for 30 minutes. Next, 50U/ml of benzonase (to degrade DNA contaminants) and 1mM of $MgCl_2$ were added and incubated at 37°C while shaking at 1500 rpm for 30 minutes. Then, 4% NaPTA and 130 mM MgCl2 were added, resulting in a final NaPTA concentration of 0.3%. The samples were incubated at 37°C while shaking at 1500 rpm for 30 minutes, followed by centrifugation at 16,000 g for 45 minutes to precipitate PrP^{Sc}. The resulting pellets were resuspended in 30 μl of 0.1% sarcosyl. 20 μl were next used for the PK-WB and digested with 20 μg ml^{-1} proteinase K for

1 hour at 37 °C. The enzymatic reaction was blocked by stop-buffer (1% (w/v) SDS; 25 mM Tris/HCl, pH 7.4; 2,5% (v/v) β-mercaptoethanol; 1.5% (w/v) sucrose; 0.02% (w/v) brome-phenol-blue). At this point, samples were boiled for 10 min prior to Western blot analysis. NBH and RML6 brain tissue underwent the same NaPTA procedure and used as negative and positive controls. Mouse monoclonal antibody against PrP (POM1, 1:5,000, homemade) was used for prions detection.

Material and Methods 'New section' - *RT-QuiC protocol.*

RT-QuIC assays were conducted as previously described (11). In brief, 10% wt/vol spleen homogenates (0.5% wt/vol sodium deoxycholate and 0.5% vol/vol Nonidet P-40 in PBS) were diluted 2000-fold and used as seeds for the RT-QuIC reactions. For skeletal muscle analysis, 2 µl of the NaPTA prion precipitation product from a 10% wt/vol sample, resuspended in 30 µl of 0.1% sarcosyl-PBS, were used. Recombinant hamster PrP (HaPrP) served as the monomeric substrate for RT-QuIC conversion. The reactions contained HaPrP substrate protein at a final concentration of 0.1 mg/ml in PBS (pH 7.4), 170 mM NaCl, 10 µM EDTA, and 10 µM Thioflavin T, with 2 µl of diluted brain homogenates added to a total volume of 100 µl. NBH and RMl6 brain homogenates were used as negative and positive, respectively.

The RT-QuIC reactions consisted of 100 hours with intermittent shaking cycles set at 42°C: 90 seconds of shaking at 900 rpm in double orbital mode, followed by 30 seconds of rest, using a FLUOstar Omega microplate reader (BMG Labtech). Thioflavin T fluorescence was measured every 15 minutes to monitor aggregate formation (450 nm excitation, 480 nm emission; bottom read mode).

Inclusion of prion strain with limited extraneural replication. Reliance on three prion strains limits the relevance. Inclusion of a strain with limited extraneural replication is suggested.

Response: Prion strains with restricted extraneural replication have not been described in mice used as prion animal models. According to our knowledge and the existing literature, there is no documentation of any mouse-adapted prion strains that are unable to propagate prions outside the CNS. We have asked several experienced colleagues, including Profs. Neil Mabbott, Jason Bartz and Joel Watts, and they all confirmed that no murine prion strain, to their knowledge, eschews replication at extraneural sites.

Although this does not apply to our study, it is important to note that hamster-derived prion strains such as HY and DY exhibit different replication patterns. Hamsters infected with HY TME prions show detectable infectivity and/or PrP^{Sc} in the CNS, lymphoreticular system, skeletal muscle,

nasal secretions, and blood (12-15). Conversely, prion infectivity and/or PrP^{Sc} in DY TME-infected hamsters is restricted to the CNS (16-18).

Nonetheless, our observations indicate that mice infected with the RML6 prion strain and sacrificed at 8 wpi show no presence of prions, whereas those infected with the ME7 and 22L strains already exhibit prion deposits in skeletal muscle (revised article Supplementary Figure 4A). This finding suggests a slower prion invasion into skeletal muscles in RML6-infected mice. Notably, Glul upregulation was already evident at 4 wpi in RML6-infected animals (revised article Supplementary Figure 7D), suggesting it might originate as a secondary consequence of brain infection or from other affected organs rather than from prions in the skeletal muscles.

Revision:

Added part in the Discussion - While prions were undetectable in skeletal muscles at early stages in mice infected with the RML6 prion strain, Glul upregulation was already evident, suggesting it might originate as a secondary consequence of brain infection or from other affected organs. Despite the presence of prions in the skeletal muscles of mice infected with ME7 and 22L prion strains, Glul upregulation was milder than that seen with RML6 prions, supporting the hypothesis that such upregulation is not directly caused by on-site prions in skeletal muscle.

Clarification on statistical methods. Lack of details on statistical tests used for comparing GLUL levels in Figures 3 and 4.

Response: We clarified the statistical tests used, specifying whether they are parametric or non-parametric. It is important to note that GLUL upregulation is significant, as evident from Figure 4. At 8 wpi, the fold change for RML6 is above 3, for ME7 is above 1.5, and for 22L is above 2. The fold change in later timepoints is increasingly larger.

Revision:

Material and Methods 'New section' - *Statistical analysis.*

For Figure 3E, normalized raw counts for the GLUL gene in control and sCJD patients were analyzed using the DESeq2 package, with related false discovery rate (FDR) calculations. In Figure 3G, Western blot densitometry data were analyzed using the Mann-Whitney U test, resulting in a p-value of 0.01072. For statistical analyses in Figure 4A, Mann-Whitney U test was used. In Figure 4C and Supplementary Figure 8 (B-C and E), we applied the t-test (as the standard deviations were consistent) with Bonferroni correction to account for multiple comparisons. In Supplementary Figure 9, the Mann-Whitney U test was used due to its non-parametric nature, which is suitable for data that do not assume a normal distribution.

The choice of DESeq2 for RNA-seq data, Mann-Whitney U for non-normally distributed data, and t-tests with Bonferroni correction for normally distributed data ensures that our analyses are appropriately tailored to the characteristics of the data, providing reliable and valid results.

Details on sCJD cases analyzed. Information regarding the types of CJD analyzed is missing.

Response: We ensured that details on the types of sCJD cases analyzed, including genotype, strain type, and age, are clearly presented in the supplementary materials. A comprehensive description of the sCJD cases, including genotype, strain type, and age, is accessible in Supplementary Table 6. This table also provides the reasons why some biosamples were excluded from the final bulk RNA sequencing and downstream analysis.

Revision: no revisions applied for this section.

Recent publications on prion seeding activity. Mention recent publications showing prion seeding activity in extraneural tissues in the Introduction.

Response: We updated the Introduction section including references to recent studies demonstrating prion seeding activity in extraneural tissues of sCJD and vCJD patients using RT-QuIC or PMCA assays.

Revision:

Added part in the Introduction - The widespread expression of PrP^{C} enables PrP^{Sc} propagation at multiple sites in prion infections. Prions can enter the body through the gastrointestinal system and accumulate in lymphoid tissue, leading to neuroinvasion via peripheral nerves (19, 20). Prion seeds are present in the spleen long before the onset of clinical symptoms (4, 21, 22). PrP^{Sc} can also be present in the blood, where it binds to plasminogen (23). Consequently, blood is a documented route of infection and a significant challenge for transfusion medicine (24, 25). PrP^{Sc} can be detected in blood with Protein Misfolding Cyclic Amplification (PMCA) as early as 2 months after inoculation (26, 27). However, PMCA is not as sensitive for detecting sporadic CJD (sCJD) (28, 29), which aligns with findings that prion transmission through blood transfusion was reported for vCJD but not for sCJD (30).

Muscle tissue has been a focal point in PrDs due to the potential for significant dietary exposure to prions through meat consumption (31, 32). This concern arises from the potential for prions to be present in muscle tissue, thereby posing a risk of transmission through the food supply. Skeletal muscles of patients with acquired and sporadic CJD show PrP^{Sc} deposits in peripheral nerve fibers (33). Unlike other organs, prions in skeletal muscle are found only late in disease (4,

10). These findings suggest that the pathogenic processes are systemic and not confined to the brain, providing possible sources for early detection.

Using PrP^{Sc} as a biomarker for early diagnosis of PrDs faces several challenges. For one, the levels of PrP^{Sc} at early disease stages are often too low for detection. Furthermore, distinct prion strains can have unique pathobiological characteristics that influence their presence in peripheral tissues and body fluids (34).

Reviewer #2 Comment:

The RNA sequencing of human skeletal muscle samples identified only one common gene between human and mouse conditions. There is concern that this gene may be a bystander result of terminal disease stage pathophysiology in both animals and human.

Response: It is important to note that the genes we intersected with sCJD bulk RNA sequencing from skeletal muscles underwent strict filtering steps. As a result, we identified only 18 genes (hub genes of the orange and darkgreen modules identified through weighted gene co-expression network analysis (WGCNA) in both the main and validation cohorts), as highlighted in Figure 3B and C. The fact that only one gene from the skeletal muscle of prion-infected animals overlaps with sCJD DEGs is due to this stringent filtering, which limited the overlap to these 18 genes.

The upregulation of GLUL cannot be attributed to a bystander effect result of terminal disease stage pathophysiology, as it is consistently upregulated across all analyzed timepoints in mice. We performed WGCNA grouped by disease stages and GLUL belongs to the orange module (upregulated genes throughout all timestages in both main (Figure 2A – revised article) and validation (Figure 3A – revised article) cohort). We also included a comparison of GLUL expression between RML6 and NBH. As shown in the new panel (revised manuscript Supplementary Figure 7D), GLUL is upregulated at all individual timepoints. This finding is corroborated at both the transcriptional and protein levels, including other prion strains (Figure 4 – revised article) from a further animal cohort for RML6 condition.

Revision:

Added part in the Result section named '*Upregulation of glutamate-ammonia ligase in skeletal muscles of human and murine prion-diseases*': We also re-evaluated the upregulation of *Glul* across all individual timepoints in skeletal muscles of prion-infected mice, noting that it remains consistently upregulated. The only exception was at timepoint 12 wpi, where the upregulation does not exceed $Log_2FC > 0.5$ (Supplementary Fig. 5D).

Cautious interpretation of GLUL dysregulation specificity. The claim that GLUL dysregulation is specific to prion diseases should be mentioned more cautiously due to the small sample number of other neurodegenerative diseases (NDs). The finding would be stronger if a meta-analysis of possible available data from human ND cohorts could be examined.

Response: We included our conclusion to acknowledge the limitation of our sample size and recommend further studies in humans for confirmation. However, due to the limited availability of skeletal muscle biopsies from other human neurodegenerative diseases, a meta-analysis cannot be performed.

Revision:

Added part in the Discussion - The identification of a common pathological phenotype among these diseases would be a significant finding, shedding light on the underlying mechanisms specific to these conditions at early stages. It is important to note that, despite the limited availability of skeletal muscle specimens from patients with neurodegenerative diseases, this phenomenon appears to be unique to prion diseases and does not occur in other common neurodegenerative disorders such as ALS, AD, or DLB.

Post-transcriptional modifications of GLUL. Explore the possibility of GLUL being modified through RNA editing affecting its expression.

Response: We investigated the potential post-transcriptional modifications of GLUL, such as RNA editing, and their impact on its expression and function.

Revision:

Results 'New section' - *Lack of post-transcriptional changes in extra-neural organs of prion-inoculated mice.*

We next calculated the genome-wide adenosine-to-inosine editing index (AEI) to measure global RNA editing levels (35), the preferential site of RNA editing in mammals. Global editing levels in blood rose steadily during aging but were independent of prion inoculation (Supplementary Fig. 2A). No AEI differences were seen in muscle or spleen (Supplementary Fig. 2, B and C). To determine recoding of individual transcripts, we aligned our sequencing results to previously published high-confidence AEI recoding sites (36). We found *Flnb* and *Copa* in the spleen and *Cog3* in blood to be significantly recoded (Supplementary Fig. 2D).

Alternative splicing can give rise to disease-associated differentially used transcripts (37). In contrast to our previous results in the brain (38), the present alternative splicing analyses in extraneural organs showed only minor alterations (Supplementary Fig. 2E). *Necap2*, *Myl6* and

Srsf5 transcripts were alternatively spliced across multiple organs and prion incubation times (Supplementary Table 4). Only in two out of a total of 21 splice variants differential transcript usage was accompanied by differential gene expression: upregulation of *Myl6* in blood at 4 wpi and downregulation of *Ms4a6c* in blood at 14 wpi.

Added part in the Discussion: Except for *Flnb*, *Copa* and *Cog3*, we were unable to find evidence for broad dysregulation of posttranscriptional RNA editing, in contrast a recent report (39) but in line with our previous findings (38). Furthermore, splicing analysis suggests that alternative splicing was largely unlinked from gene expression changes.

Material and Methods 'New section': *Post-trascriptional changes analysis.*

Adenosine-to-inosine editing index (AEI) was calculated as previously published (35). Herein, raw fastq reads were uniquely aligned to a murine mm10 reference genome using STAR v2.7.3 with the filter outFilterMultimapNmax=1. RNAEditingIndexer (https://github.com/a2iEditing/RNAEditingIndexer) was used to calculate per-sample AEI.

We identified gene-specific RNA editing based on a recently published list of high-confidence targets of *Adar* (36) as follows. RediToolsKnown.py from REDItools (40) was applied on uniquely aligned samples as mentioned above. This yielded per-site lists of A-to-I editing on which we applied the following thresholds: (a) a minimum of 3 alternative reads per site per sample (b) a minimal editing frequency of 1 % per site (c) criteria a) and b) are fulfilled in at least floor(2/3 * n) biological replicates, n is total number of biological replicates per group (d) transcripts of site present in at least 2 biological control replicates. Multiple testing of sites passing above-mentioned thresholds was performed using REDIT (https://github.com/gxiaolab/REDITs) and adjusted for false discovery rate (FDR) according to Benjamini-Hochberg, we considered sites with an FDR < 0.05 to be significantly edited.

For alternative splicing, SGSeq R package (41) was employed to find splicing events characterized by two or more splice variants. Exons and splice junction predictions were obtained from BAM filesPrediction of exons and splice junctions was first made for each sample individually. Then the predictions for all samples were merged and we obtained a common set of transcript features. Overlapping exons were disjoint into non-overlapping exon bins and a genome-wide splice graph was compiled based on splice junctions and exon bins. A single value for each variant was produced by adding up the 5' and 3' counts, or, if these represented the same transcript features, by considering the unique value. These counts were then fed to DEXSeq (42). We analyzed differential usage of variants across a single event, in-stead of quantifying differential usage of exons across a single gene. We retained only variants with at least five counts

in at least three samples (of any condition). After filtering, the events associated with a single variant were discarded. Differential analysis was then performed implementing a sample+exon+condition:exon model in DEXSeq. Differentially expressed isoforms were defined as isoforms changing with FDR < 0.05. In the case of differentially used splice variants in muscle on 12 wpi, this dataset was considered as an outlier and hence excluded due to excessively reported splice variants (1,788 events compared to 5 or less on all other time-points and extraneural organs).

References

1. Sorce S, Nuvolone M, Russo G, Chincisan A, Heinzer D, Avar M, et al. Genome-wide transcriptomics identifies an early preclinical signature of prion infection. PLoS Pathog. 2020;16(6):e1008653.
2. Foutz A, Appleby BS, Hamlin C, Liu X, Yang S, Cohen Y, et al. Diagnostic and prognostic value of human prion detection in cerebrospinal fluid. Annals of neurology. 2017;81(1):79-92.
3. Cramm M, Schmitz M, Karch A, Mitrova E, Kuhn F, Schroeder B, et al. Stability and reproducibility underscore utility of RT-QuIC for diagnosis of Creutzfeldt-Jakob disease. Molecular neurobiology. 2016;53:1896-904.
4. Shi S, Mitteregger-Kretzschmar G, Giese A, Kretzschmar HA. Establishing quantitative real-time quaking-induced conversion (qRT-QuIC) for highly sensitive detection and quantification of PrPSc in prion-infected tissues. Acta Neuropathologica Communications. 2013;1(1):44.
5. Brown KL, Stewart K, Ritchie DL, Mabbott NA, Williams A, Fraser H, et al. Scrapie replication in lymphoid tissues depends on prion protein-expressing follicular dendritic cells. Nature medicine. 1999;5(11):1308-12.
6. Mabbott NA, Williams A, Farquhar CF, Pasparakis M, Kollias G, Bruce ME. Tumor necrosis factor alpha-deficient, but not interleukin-6-deficient, mice resist peripheral infection with scrapie. Journal of virology. 2000;74(7):3338-44.
7. Glatzel M, Abela E, Maissen M, Aguzzi A. Extraneural pathologic prion protein in sporadic Creutzfeldt-Jakob disease. The New England journal of medicine. 2003;349(19):1812-20.
8. Wells GA, Hawkins SA, Green RB, Austin AR, Dexter I, Spencer YI, et al. Preliminary observations on the pathogenesis of experimental bovine spongiform encephalopathy (BSE): an update. The Veterinary record. 1998;142(5):103-6.
9. Wilham JM, Orrú CD, Bessen RA, Atarashi R, Sano K, Race B, et al. Rapid end-point quantitation of prion seeding activity with sensitivity comparable to bioassays. PLoS Pathog. 2010;6(12):e1001217.
10. Neumann M, Krasemann S, Schröck K, Steinbach K, Glatzel M. Myositis facilitates preclinical accumulation of pathological prion protein in muscle. Acta Neuropathol Commun. 2013;1(1):78.
11. Pfammatter M, Andreasen M, Meisl G, Taylor CG, Adamcik J, Bolisetty S, et al. Absolute quantification of amyloid propagons by digital microfluidics. Analytical chemistry. 2017;89(22):12306-13.
12. Bessen RA, Shearin H, Martinka S, Boharski R, Lowe D, Wilham JM, et al. Prion shedding from olfactory neurons into nasal secretions. PLoS pathogens. 2010;6(4):e1000837.
13. Mulcahy ER, Bartz JC, Kincaid AE, Bessen RA. Prion infection of skeletal muscle cells and papillae in the tongue. Journal of virology. 2004;78(13):6792-8.
14. Marsh R, Bessen R. Physicochemical and biological characterizations of distinct strains of the transmissible mink encephalopathy agent. Philosophical Transactions of the Royal Society of London Series B: Biological Sciences. 1994;343(1306):413-4.
15. Bessen RA, Marsh RF. Identification of two biologically distinct strains of transmissible mink encephalopathy in hamsters. Journal of General Virology. 1992;73(2):329-34.
16. Bessen RA, Martinka S, Kelly J, Gonzalez D. Role of the lymphoreticular system in prion neuroinvasion from the oral and nasal mucosa. Journal of virology. 2009;83(13):6435-45.

17. Bartz JC, Aiken JM, Bessen RA. Delay in onset of prion disease for the HY strain of transmissible mink encephalopathy as a result of prior peripheral inoculation with the replication-deficient DY strain. Journal of general virology. 2004;85(1):265-73.
18. Bartz JC, Kincaid AE, Bessen RA. Rapid prion neuroinvasion following tongue infection. Journal of virology. 2003;77(1):583-91.
19. Klein MA, Frigg R, Flechsig E, Raeber AJ, Kalinke U, Bluethmann H, et al. A crucial role for B cells in neuroinvasive scrapie. Nature. 1997;390(6661):687-90.
20. Mabbott NA, Mackay F, Minns F, Bruce ME. Temporary inactivation of follicular dendritic cells delays neuroinvasion of scrapie. Nature medicine. 2000;6(7):719-20.
21. Béringue V, Tixador P, Andréoletti O, Reine F, Castille J, Laï TL, et al. Host prion protein expression levels impact prion tropism for the spleen. PLoS Pathog. 2020;16(7):e1008283.
22. Chen B, Soto C, Morales R. Peripherally administrated prions reach the brain at sub-infectious quantities in experimental hamsters. FEBS Letters. 2014;588(5):795-800.
23. Fischer MB, Roeckl C, Parizek P, Schwarz HP, Aguzzi A. Binding of disease-associated prion protein to plasminogen. Nature. 2000;408(6811):479-83.
24. Houston F, Foster JD, Chong A, Hunter N, Bostock CJ. Transmission of BSE by blood transfusion in sheep. Lancet. 2000;356(9234):999-1000.
25. Salamat MKF, Blanco ARA, McCutcheon S, Tan KB, Stewart P, Brown H, et al. Preclinical transmission of prions by blood transfusion is influenced by donor genotype and route of infection. PLoS pathogens. 2021;17(2):e1009276.
26. Concha-Marambio L, Chacon MA, Soto C. Preclinical Detection of Prions in Blood of Nonhuman Primates Infected with Variant Creutzfeldt-Jakob Disease. Emerg Infect Dis. 2020;26(1):34-43.
27. Concha-Marambio L, Pritzkow S, Moda F, Tagliavini F, Ironside JW, Schulz PE, et al. Detection of prions in blood from patients with variant Creutzfeldt-Jakob disease. Science translational medicine. 2016;8(370):370ra183.
28. Moda F, Gambetti P, Notari S, Concha-Marambio L, Catania M, Park K-W, et al. Prions in the Urine of Patients with Variant Creutzfeldt–Jakob Disease. New England Journal of Medicine. 2014;371(6):530-9.
29. Bougard D, Brandel J-P, Bélondrade M, Béringue V, Segarra C, Fleury H, et al. Detection of prions in the plasma of presymptomatic and symptomatic patients with variant Creutzfeldt-Jakob disease. Science translational medicine. 2016;8(370):370ra182-370ra182.
30. Llewelyn CA, Hewitt PE, Knight RS, Amar K, Cousens S, Mackenzie J, et al. Possible transmission of variant Creutzfeldt-Jakob disease by blood transfusion. Lancet. 2004;363(9407):417-21.
31. Prusiner SB. Prion biology and diseases1999.
32. Stärk KDC, Regula G, Hernandez J, Knopf L, Fuchs K, Morris RS, et al. Concepts for risk-based surveillance in the field of veterinary medicine and veterinary public health: Review of current approaches. BMC Health Services Research. 2006;6(1):20.
33. Peden AH, Ritchie DL, Head MW, Ironside JW. Detection and localization of PrPSc in the skeletal muscle of patients with variant, latrogenic, and sporadic forms of Creutzfeldt-Jakob disease. American Journal of Pathology. 2006;168(3):927-35.
34. Herzog C, Rivière J, Lescoutra-Etchegaray N, Charbonnier A, Leblanc V, Salès N, et al. PrP^{TSE} Distribution in a Primate Model of Variant, Sporadic, and Iatrogenic Creutzfeldt-Jakob Disease. Journal of virology. 2005;79(22):14339-45.
35. Roth SH, Levanon EY, Eisenberg E. Genome-wide quantification of ADAR adenosine-to-inosine RNA editing activity. Nature methods. 2019;16(11):1131-8.
36. Gabay O, Shoshan Y, Kopel E, Ben-Zvi U, Mann TD, Bressler N, et al. Landscape of adenosine-to-inosine RNA recoding across human tissues. Nat Commun. 2022;13(1):1184.

37. Scotti MM, Swanson MS. RNA mis-splicing in disease. Nat Rev Genet. 2016;17(1):19-32.
38. Sorce S, Nuvolone M, Russo G, Chincisan A, Heinzer D, Avar M, et al. Genome-wide transcriptomics identifies an early preclinical signature of prion infection. PLoS Pathog. 2020;16(6):e1008653.
39. Kanata E, Llorens F, Dafou D, Dimitriadis A, Thune K, Xanthopoulos K, et al. RNA editing alterations define manifestation of prion diseases. Proc Natl Acad Sci U S A. 2019;116(39):19727-35.
40. Picardi E, Pesole G. REDItools: high-throughput RNA editing detection made easy. Bioinformatics. 2013;29(14):1813-4.
41. Goldstein LD, Cao Y, Pau G, Lawrence M, Wu TD, Seshagiri S, et al. Prediction and Quantification of Splice Events from RNA-Seq Data. PloS one. 2016;11(5):e0156132.
42. Anders S, Reyes A, Huber W. Detecting differential usage of exons from RNA-seq data. Genome Res. 2012;22(10):2008-17.
