# Supplementary material for: Prion diseases disrupt glutamate/glutamine metabolism in skeletal muscle
Source: PLoS Pathog. 2024 Sep 11;20(9):e1012552. doi: 10.1371/journal.ppat.1012552 (PMC11419395; doi:10.1371/journal.ppat.1012552)
Supplement: Attachment — Submitted filename: 145824_1_rebuttal_3042061_s7kh23.docx [file ppat.1012552.s023.docx]

**Manuscript number:** RC-2024-02456

**Corresponding author(s):** Adriano, Aguzzi

## 1. General Statements

*This section is optional. Insert here any general statements you wish to make about the goal of the study or about the reviews.*

In this study, our primary objective was to investigate the pathophysiological changes occurring in various extraneural organs during prion disease progression. Our focus was on understanding the broader biological impacts of prion infections, particularly alterations in molecular pathways such as the observed glutamine-glutamate cycle. Our expectation was that this approach may provide a comprehensive understanding of the disease’s systemic effects and identifies potential biomarkers and therapeutic targets relevant to prion diseases.

Our study has generated a FAIR-compliant (Findable, Accessible, Interoperable, Reusable) longitudinal atlas of transcriptional alterations in the CNS level and in an extensive ensemble of extraneural organs. This broader perspective is crucial for developing a more holistic understanding of prion diseases.

Detecting prion presence in organs like skeletal muscles does not directly imply that the observed GLUL and transcriptomic changes are due to prions on site. As suggested by Reviewer #1, addressing this question would require using prion strains that do not spread outside the brain, whose existence is questionable. Such an experiment may (or may not) clarify whether the alteration in the glutamine-glutamate cycle is a result of prion spreading in the brain, which is not the question addressed in our study.

## 2. Description of the planned revisions

*Insert here a point-by-point reply that explains what revisions, additional experimentations and analyses are planned to address the points raised by the referees.*

**Reviewer #1 Comment:**

*Absence of evidence demonstrating the presence of prions or prion-seeding activity in extraneural tissues*.

Response: Our study focuses on *all* pathophysiological alterations brought about by prion infections in extraneural organs, be they caused directly by prion infections of said organs or through indirect mechanisms. Nevertheless, we share the reviewer’s curiosity about a possible correlation between these changes and the presence of prion-seeding activity. We propose to include additional data demonstrating prion presence and seeding activity in skeletal muscle at various timepoints. This will be achieved by employing proteinase K digestion followed by Western blot (PK-WB) and real-time quaking-induced conversion (RT-QuIC) assays to provide a robust correlation with our transcriptomic analyses and Glul upregulation.

Revision: We have conducted preliminary experiments using PK-WB and RT-QuIC assays. These experiments were performed on terminal-stage prion-infected samples and related controls. If prion presence is detected at this stage, we plan to extend the analysis to earlier stages, specifically at 16 weeks post-inoculation (wpi) and 8 wpi, to track the progression of prion-seeding activity over time.

Western blot analysis was performed on skeletal muscle homogenates at the terminal stage of prion disease in mice infected with three prion strains (RML6, ME7, and 22L) and related NBH control. Control samples include CNS brain homogenates, showing prion presence (PrP^Sc^) in RML6 but not in NBH. Neither skeletal muscles from NBH nor from prion-infected samples show detectable prions, indicating that PK-WB may lack the sensitivity to detect prions in skeletal muscle or that prion levels are below detection thresholds (**Revision Figure 1A**) for this specific technique.

Next, we performed RT-QuIC assays on muscle homogenates using different protocols, including sodium phosphotungstic acid (NaPTA) enrichment (**Revision Figure 1B**). NaPTA binds and precipitates PrP^Sc^ in the presence of MgCl_2_, removing contaminants and concentrating PrP^Sc^. For this protocol, 100 µg of muscle homogenates were treated with benzonase to degrade DNA contaminants. Then, 4% NaPTA and 170 mM MgCl2 were added, resulting in a final NaPTA concentration of 0.3%. The samples were incubated at 37°C while shaking at 1500 rpm for 2 hours, followed by centrifugation at 15,000 g for 30 minutes to precipitate PrP^Sc^. The resulting pellets were used to seed RT-QuIC reactions. Each biological replicate was run in quadruplicate, and replicates were considered positive only if at least 3 out of 4 technical replicates showed detection. Results indicated no amplification for NBH samples. For RML6 skeletal muscles, 2 out of 3 biological replicates were positive. For ME7, 0 out of 4 biological replicates were positive. For 22L, 1 out of 3 biological replicates was positive.

Using the same protocol without NaPTA (**Revision Figure 1C**), two positive samples were observed in the NBH condition, suggesting that NaPTA is useful for specific prion enrichment.

Similarly, we combined NaPTA and sarkosyl in our further trial. 10% weight/volume muscle homogenates in 1x PBS containing 2% sarkosyl were centrifuged at 80 g for 1 minute, and the supernatant (500 µl) was collected. If still dirty, further centrifugation at 2700 g for 5 minutes was performed. Then, 500 µl PBS containing 2% sarkosyl was added and incubated for 10 minutes at 37°C. Benzonase and MgCl2 (final concentrations 50 U/ml and 1 mM, respectively) were added and incubated for 30 minutes at 37°C with shaking at 1500 rpm. NaPTA was added to a final concentration of 0.3% and incubated for 30 minutes at 37°C with shaking. Samples were centrifuged at 15,000 g for 30 minutes and resuspended in 20 µl of 0.1% sarkosyl-containing PBS condition (**Revision Figure 1D**). Using this protocol, we also detected signals in the NBH control.

These results indicate ongoing challenges in optimizing prion extraction from skeletal muscle. Unlike brain tissue, where prion levels are significantly higher, skeletal muscle presents difficulties due to lower prion concentrations. In brain samples, dilutions still result in positive signals only from prion-enriched conditions. However, for skeletal muscle, prion extraction is not as straightforward, highlighting the need for further refinement of the protocol to achieve reliable detection and differentiation between prion-infected and control samples.

*Inclusion of prion strain with limited extraneural replication. Reliance on three prion strains limits the relevance. Inclusion of a strain with limited extraneural replication is suggested*.

Response: To address this limitation, we propose a comprehensive discussion on the systemic nature of prion diseases, emphasizing the need for future research to explore potential strains with restricted replication patterns.

Revision: There is a significant interest in prion deposits in skeletal muscles as potential sources of prion spreading. The consumption of beef products from cattle infected with bovine spongiform encephalopathy (BSE) prions caused new variant Creutzfeldt–Jakob disease, raising early concerns about the transmission of prions from cervids to humans (1-3). This remains a hot topic in the field (4-7), underscoring the importance of our longitudinal transcriptomic analysis in different extraneural organs. However, prion strains with restricted extraneural replication which we could use as control have not been described in mice used as prion animal models. According to our knowledge and the existing literature, there is no documentation of any mouse-adapted prion strains that are unable to propagate prions outside the central nervous system (CNS). Although this does not apply to our study, it is important to note that hamster-derived prion strains such as HY and DY exhibit different replication patterns. Hamsters infected with HY TME prions show detectable infectivity and/or PrP^Sc^ in the CNS, lymphoreticular system, skeletal muscle, nasal secretions, and blood (8-11). Conversely, prion infectivity and/or PrP^Sc^ in DY TME-infected hamsters is restricted to the CNS (12-14).

In mice, the situation is quite different, and there are no prion strains with restricted extraneural replication. Instead, studies have focused on models where prion protein (PrP^C^) is absent in all tissues except skeletal muscle, which is essentially the opposite of the condition requested by the reviewer. For instance, research has demonstrated that prion levels in skeletal muscles are 5-10% of those observed in the brain (1). Here, transgenic mice (Tg(α-actin-MoPrP)6906/Prnp^0/0^) expressing PrP^C^ only in skeletal muscles (and barely detectable in the CNS) were created. After intramuscular prion injection, these mice showed that skeletal muscles could propagate prions (1). Additionally, another study found that prions were not detectable in skeletal muscle at early stages (32 and 60 days post intracerebral prion inoculation) unless experimental autoimmune myositis was induced, which increased prion spread to skeletal muscle (15).

This comprehensive discussion underscores the absence of a mouse model prion strain with limited extraneural replication, highlighting a gap in current research that our study aims to address indirectly through our systemic approach.

*Clarification on statistical methods. Lack of details on statistical tests used for comparing GLUL levels in Figures 3 and 4*.

Response: We clarified the statistical tests used, specifying whether they are parametric or non-parametric, and provide a rationale for the chosen methods. It is important to note that GLUL upregulation is significant, as evident from Figure 4. At 8 wpi, the fold change for RML6 is above 3, for ME7 is above 1.5, and for 22L is above 2. The fold change in later timepoints is increasingly larger.

Revision: For Figure 3E, normalized raw counts for the GLUL gene in control and sCJD patients were analyzed using the DESeq2 package, with related false discovery rate (FDR) calculations. DESeq2 is appropriate for RNA-seq data as it models count data using a negative binomial distribution, suitable for overdispersed count data commonly found in RNA-seq experiments. The normalization and FDR calculation ensure that the comparisons between control and sCJD patients are statistically robust.

In Figure 3G, Western blot densitometry data were analyzed using the Mann-Whitney U test, resulting in a p-value of 0.01072. The Mann-Whitney U test is a non-parametric test that does not assume normal distribution, making it suitable for small sample sizes and non-normally distributed data, which is often the case in Western blot densitometry. For statistical analyses in Figure 4A, Mann-Whitney U test was used. In Figure 4C, we applied the t-test (as the standard deviations were consistent) with Bonferroni correction to account for multiple comparisons. The t-test is a parametric test suitable for normally distributed data, and the Bonferroni correction adjusts for the increased risk of Type I errors when multiple comparisons are made, ensuring the results are not due to chance. Additionally, we used one-way ANOVA corrected with Kruskal-Wallis, a non-parametric method, to confirm our findings (**Revision Figure 2**). The results from both statistical tests were in strong agreement, validating our analysis.

In Supplementary Figure 6, the Mann-Whitney U test was used due to its non-parametric nature, which is suitable for data that do not assume a normal distribution. This test was chosen to provide a robust analysis of the data, which did not fit the assumptions required for parametric tests.

These methods were selected based on the data distribution and the need for accurate statistical analysis to validate the significance of our findings. The choice of DESeq2 for RNA-seq data, Mann-Whitney U for non-normally distributed data, and t-tests with Bonferroni correction for normally distributed data ensures that our analyses are appropriately tailored to the characteristics of the data, providing reliable and valid results.

*Details on CJD cases analyzed. Information regarding the types of CJD analyzed is missing*.

Response: We ensured that details on the types of CJD cases analyzed, including genotype, strain type, and age, are clearly presented in the main text and supplementary materials.

Revision: A comprehensive description of the sCJD cases, including genotype, strain type, and age, was accessible in Supplementary Table 5 (and here, in **Revision Figure 3**). This table also provides the reasons why some biosamples were excluded from the final bulk RNA sequencing and downstream analysis.

*Recent publications on prion seeding activity. Mention recent publications showing prion seeding activity in extraneural tissues*.

Response: We will update the Introduction section to include references to recent studies demonstrating prion seeding activity in extraneural tissues of sCJD and vCJD patients using RT-QuIC or PMCA assays.

Revision: We will discuss and cite additional papers on this topic, highlighting the growing body of evidence for prion seeding activity in extraneural tissues. These references will provide a comprehensive background on the detection and significance of prion seeding in peripheral tissues, thereby strengthening the context and relevance of our study.

**Reviewer #2 Comment:**

*The RNA sequencing of human skeletal muscle samples identified only one common gene between human and mouse conditions. There is concern that this gene may be a bystander result of terminal disease stage pathophysiology in both animals and human*.

Response: We strengthened the evidence supporting GLUL as early and altered gene by including additional timepoint analyses and showing its presence at earlier disease stages.

Revision: The upregulation of GLUL cannot be attributed to a bystander effect result of terminal disease stage pathophysiology, as it is consistently upregulated across all analyzed timepoints. We performed weighted gene co-expression network analysis (WGCNA) grouped by disease stages and GLUL belongs to the orange module (upregulated genes throughout all timestages in both main (Figure 2A – original manuscript) and validation (Figure 3A – original manuscript) cohort). We also included a comparison of GLUL expression between RML6 and NBH. As shown in the figure (**Revision Figure 4**), GLUL is upregulated at all individual timepoints. This finding is corroborated at both the transcriptional and protein levels, including other prion strains (Figure 4 – original manuscript) from a further animal cohort for RML6 condition. This consistent upregulation across various stages supports GLUL as a robust altered genes and possible biomarker for prion disease progression.

*Cautious interpretation of GLUL dysregulation specificity. The claim that GLUL dysregulation is specific to prion diseases should be mentioned more cautiously due to the small sample number of other neurodegenerative diseases (NDs). The finding would be stronger if a meta-analysis of possible available data from human ND cohorts could be examined.*

Response: We will rephrase our conclusion to acknowledge the sample size limitation and suggest further studies for confirmation. However, due to the poor sample availability of skeletal muscles biopsis from other NDs, related metadata are complicated to be found.

Revision: Modify the discussion section to reflect a more cautious interpretation, emphasizing the need for larger cohort studies to confirm GLUL specificity.

*Post-transcriptional modifications of GLUL. Explore the possibility of GLUL being modified through RNA editing affecting its expression.*

Response: We investigated the potential post-transcriptional modifications of GLUL, such as RNA editing, and their impact on its expression and function.

Revision: We will add a paragraph named “*Lack of post-transcriptional changes in extra-neural organs of prion-inoculated mice*”.

**Results**: We calculated the genome-wide adenosine-to-inosine editing index (AEI) to measure global RNA editing levels (16), the preferential site of RNA editing in mammals. Blood global editing levels rose steadily during aging but were independent of prion inoculation (**Revision Figure 5A**). No AEI differences were seen in muscle or spleen (**Revision Figure 5B and C**). To determine recoding of individual transcripts, we aligned our sequencing results to previously published high-confidence AEI recoding sites (17). We found *Flnb* and *Copa* in the spleen and *Cog3* in blood to be significantly recoded (**Revision Figure 5D**). However, Glul did not show significant recoding.

Alternative splicing can give rise to disease-associated differentially used transcripts (18). In contrast to our previous results in the brain (19), the present alternative splicing analyses in extraneural organs showed only minor alterations (**Revision Figure 5E**). *Necap2*, *Myl6* and *Srsf5* transcripts were alternatively spliced across multiple organs and prion incubation times (**Revision Table 1**). Only in two out of a total of 21 splice variants differential transcript usage was accompanied by differential gene expression: upregulation of *Myl6* in blood at 4 wpi and downregulation of *Ms4a6c* in blood at 14 wpi.

**Discussion**: Except for *Flnb*, *Copa* and *Cog3*, we were unable to find evidence for broad dysregulation of posttranscriptional RNA editing, in contrast a recent report (20) but in line with our previous findings (19). Furthermore, splicing analysis suggests that alternative splicing was largely unlinked from gene expression changes.

**Method**: Adenosine-to-inosine editing index (AEI) was calculated as previously published (16). Herein, raw fastq reads were uniquely aligned to a murine mm10 reference genome using STAR v2.7.3 with the filter outFilterMultimapNmax=1. RNAEditingIndexer (https://github.com/a2iEditing/RNAEditingIndexer) was used to calculate per-sample AEI.

We identified gene-specific RNA editing based on a recently published list of high-confidence targets of *Adar* (17) as follows. RediToolsKnown.py from REDItools (21) was applied on uniquely aligned samples as mentioned above. This yielded per-site lists of A-to-I editing on which we applied the following thresholds: (a) a minimum of 3 alternative reads per site per sample (b) a minimal editing frequency of 1 % per site (c) criteria a) and b) are fulfilled in at least floor(2/3 * n) biological replicates, n is total number of biological replicates per group (d) transcripts of site present in at least 2 biological control replicates. Multiple testing of sites passing above-mentioned thresholds was performed using REDIT (<https://github.com/gxiaolab/REDITs>) and adjusted for false discovery rate (FDR) according to Benjamini-Hochberg, we considered sites with an FDR < 0.05 to be significantly edited.

For alternative splicing, SGSeq R package (22) was employed to find splicing events characterized by two or more splice variants. Exons and splice junction predictions were obtained from BAM filesPrediction of exons and splice junctions was first made for each sample individually. Then the predictions for all samples were merged and we obtained a common set of transcript features. Overlapping exons were disjoint into non-overlapping exon bins and a genome-wide splice graph was compiled based on splice junctions and exon bins. A single value for each variant was produced by adding up the 5’ and 3’ counts, or, if these represented the same transcript features, by considering the unique value. These counts were then fed to DEXSeq (23). We analyzed differential usage of variants across a single event, in-stead of quantifying differential usage of exons across a single gene. We retained only variants with at least five counts in at least three samples (of any condition). After filtering, the events associated with a single variant were discarded. Differential analysis was then performed implementing a sam-ple+exon+condition:exon model in DEXSeq. Differentially expressed isoforms were defined as isoforms changing with FDR < 0.05. In the case of differentially used splice variants in muscle on 12 wpi, this dataset was considered as an outlier and hence excluded due to excessively reported splice variants (1,788 events compared to 5 or less on all other time-points and extraneural organs).

## 3. Description of the revisions that have already been incorporated in the transferred manuscript

*Please insert a point-by-point reply describing the revisions that were already carried out and included in the transferred manuscript. If no revisions have been carried out yet, please leave this section empty.*

## 4. Description of analyses that authors prefer not to carry out

*Please include a point-by-point response explaining why some of the requested data or additional analyses might not be necessary or cannot be provided within the scope of a revision. This can be due to time or resource limitations or in case of disagreement about the necessity of such additional data given the scope of the study. Please leave empty if not applicable.*

We do not plan to include a cohort of animals inoculated with a prion strain that has limited extraneural spreading and deposition. This decision is based on the fact that, to our knowledge, no such strain exists. Additionally, intracerebral injection of prions could lead to spillover, mimicking an intravenous-like inoculation alongside intracerebral infection. This spillover could potentially cause alterations in the glutamine-glutamate pathway, complicating the interpretation of results. Thus, our focus remains on understanding the systemic effects of prion diseases using established prion strains.

**Revision Figure 1. A)** Western blot analysis of skeletal muscle homogenates at the terminal stage of prion disease in mice infected with prion strains (RML6, ME7, and 22L). Control CNS brain homogenates from NBH and RML6 mice are included. Prion protein (PrP^Sc^) is detected in RML6 brain but not in NBH control. No prion signal is detected in skeletal muscle samples, indicating either the insensitivity of PK-WB for prions in skeletal muscle or that prion levels are below detection limits. **B-D)** RT-QuIC assay of prion-seeding activity in muscle homogenates enriched via **B)** NaPTA precipitation, **C)** without NaPTA precipitation, and **D)** without NaPTA and enriched with Sarkosyl.

**Revision Figure 2.** **A)** Western blot images showing the expression of GLUL and Vinculin (loading control) in skeletal muscle samples from mice infected with prion strains (RML6, ME7, 22L) and control (NBH) at 8 weeks post-inoculation (wpi), 16 wpi, and terminal stage. **B)** Quantification of GLUL protein levels normalized to Vinculin in the same samples at 8 wpi, 16 wpi, and terminal stage. Data are presented as means ± standard deviations. Statistical significance was assessed using a one-way ANOVA with Kruskal-Wallis correction for multiple comparisons, with significance indicated as *p < 0.05 and **p < 0.01.

**Revision Figure 3** (Supplementary Table 5 in the current manuscript): this table contains detailed information on all the different biosamples collected from each patient. The last three columns highlight transcriptomics-related information, specifying whether and why each biosample was included or excluded from the analysis.

**Revision Figure 4.** The graph displays the log_2_ fold change of GLUL expression in skeletal muscle at various time points (4, 8, 12, 14, 16, 18, 20 weeks post-inoculation (wpi), and terminal stage). The y-axis represents the log_2_ fold change, indicating the level of GLUL upregulation relative to the control. The x-axis shows the time points post-inoculation.

**Revision Figure 5.** These panels illustrate the percentage of alternative splicing events (AEI) in blood (**A**), muscle (**B**), and spleen (**C**) samples of prion-infected (RML6) and control (NBH) mice at various weeks post-inoculation. The y-axis represents the AEI percentage, while the x-axis shows the weeks post-inoculation. **D)** Significantly recoded gene transcripts Flnb and Cog3 genes in spleen and blood, respectively (left and right panel). Reduced A-to-I editing of Copa transcripts in RML6-inoculated mice (middle panel). **E)** Bars show annotation and related number of splice variants in both extraneural organs and brains of data from Sorce and Nuvolone et al. 2020.

**References**

1.	Bosque PJ, Ryou C, Telling G, Peretz D, Legname G, DeArmond SJ, et al. Prions in skeletal muscle. *Proc Natl Acad Sci U S A.* 2002;99(6):3812-7.

2.	Belay ED, Gambetti P, Schonberger LB, Parchi P, Lyon DR, Capellari S, et al. Creutzfeldt-Jakob disease in unusually young patients who consumed venison. *Archives of neurology.* 2001;58(10):1673-8.

3.	Pattison I, and Millson G. Distribution of the scrapie agent in the tissues of experimentally inoculated goats. *Journal of comparative Pathology.* 1962;72:233-44.

4.	DeVivo MT, Edmunds DR, Kauffman MJ, Schumaker BA, Binfet J, Kreeger TJ, et al. Endemic chronic wasting disease causes mule deer population decline in Wyoming. *PloS one.* 2017;12(10):e0186512.

5.	Edmunds DR, Kauffman MJ, Schumaker BA, Lindzey FG, Cook WE, Kreeger TJ, et al. Chronic wasting disease drives population decline of white-tailed deer. *PloS one.* 2016;11(8):e0161127.

6.	Geremia C, Miller MW, Hoeting JA, Antolin MF, and Hobbs NT. Bayesian modeling of prion disease dynamics in mule deer using population monitoring and capture-recapture data. *PloS one.* 2015;10(10):e0140687.

7.	Mori J, Rivera N, Novakofski J, and Mateus-Pinilla N. A review of chronic wasting disease (CWD) spread, surveillance, and control in the United States captive cervid industry. *Prion.* 2024;18(1):54-67.

8.	Bessen RA, Shearin H, Martinka S, Boharski R, Lowe D, Wilham JM, et al. Prion shedding from olfactory neurons into nasal secretions. *PLoS pathogens.* 2010;6(4):e1000837.

9.	Mulcahy ER, Bartz JC, Kincaid AE, and Bessen RA. Prion infection of skeletal muscle cells and papillae in the tongue. *Journal of virology.* 2004;78(13):6792-8.

10.	Marsh R, and Bessen R. Physicochemical and biological characterizations of distinct strains of the transmissible mink encephalopathy agent. *Philosophical Transactions of the Royal Society of London Series B: Biological Sciences.* 1994;343(1306):413-4.

11.	Bessen RA, and Marsh RF. Identification of two biologically distinct strains of transmissible mink encephalopathy in hamsters. *Journal of General Virology.* 1992;73(2):329-34.

12.	Bessen RA, Martinka S, Kelly J, and Gonzalez D. Role of the lymphoreticular system in prion neuroinvasion from the oral and nasal mucosa. *Journal of virology.* 2009;83(13):6435-45.

13.	Bartz JC, Aiken JM, and Bessen RA. Delay in onset of prion disease for the HY strain of transmissible mink encephalopathy as a result of prior peripheral inoculation with the replication-deficient DY strain. *Journal of general virology.* 2004;85(1):265-73.

14.	Bartz JC, Kincaid AE, and Bessen RA. Rapid prion neuroinvasion following tongue infection. *Journal of virology.* 2003;77(1):583-91.

15.	Neumann M, Krasemann S, Schröck K, Steinbach K, and Glatzel M. Myositis facilitates preclinical accumulation of pathological prion protein in muscle. *Acta Neuropathologica Communications.* 2013;1(1):78.

16.	Roth SH, Levanon EY, and Eisenberg E. Genome-wide quantification of ADAR adenosine-to-inosine RNA editing activity. *Nature methods.* 2019;16(11):1131-8.

17.	Gabay O, Shoshan Y, Kopel E, Ben-Zvi U, Mann TD, Bressler N, et al. Landscape of adenosine-to-inosine RNA recoding across human tissues. *Nat Commun.* 2022;13(1):1184.

18.	Scotti MM, and Swanson MS. RNA mis-splicing in disease. *Nat Rev Genet.* 2016;17(1):19-32.

19.	Sorce S, Nuvolone M, Russo G, Chincisan A, Heinzer D, Avar M, et al. Genome-wide transcriptomics identifies an early preclinical signature of prion infection. *PLoS Pathog.* 2020;16(6):e1008653.

20.	Kanata E, Llorens F, Dafou D, Dimitriadis A, Thune K, Xanthopoulos K, et al. RNA editing alterations define manifestation of prion diseases. *Proc Natl Acad Sci U S A.* 2019;116(39):19727-35.

21.	Picardi E, and Pesole G. REDItools: high-throughput RNA editing detection made easy. *Bioinformatics.* 2013;29(14):1813-4.

22.	Goldstein LD, Cao Y, Pau G, Lawrence M, Wu TD, Seshagiri S, et al. Prediction and Quantification of Splice Events from RNA-Seq Data. *PloS one.* 2016;11(5):e0156132.

23.	Anders S, Reyes A, and Huber W. Detecting differential usage of exons from RNA-seq data. *Genome Res.* 2012;22(10):2008-17.
